# Reduced Dopamine Signaling Impacts Pyramidal Neuron Excitability in Mouse Motor Cortex

**DOI:** 10.1523/ENEURO.0548-19.2021

**Published:** 2021-10-13

**Authors:** Olivia K. Swanson, Rosa Semaan, Arianna Maffei

**Affiliations:** 1 Department of Neurobiology and Behavior, Stony Brook University, Stony Brook, New York 11794; 2 Graduate Program in Neuroscience, Stony Brook University, Stony Brook, New York 11794

**Keywords:** dopamine, excitability, modulation, motor cortex, neurodegeneration, neuron

## Abstract

Dopaminergic modulation is essential for the control of voluntary movement; however, the role of dopamine in regulating the neural excitability of the primary motor cortex (M1) is not well understood. Here, we investigated two modes by which dopamine influences the input/output function of M1 neurons. To test the direct regulation of M1 neurons by dopamine, we performed whole-cell recordings of excitatory neurons and measured excitability before and after local, acute dopamine receptor blockade. We then determined whether chronic depletion of dopaminergic input to the entire motor circuit, via a mouse model of Parkinson’s disease, was sufficient to shift M1 neuron excitability. We show that D1 receptor (D1R) and D2R antagonism altered subthreshold and suprathreshold properties of M1 pyramidal neurons in a layer-specific fashion. The effects of D1R antagonism were primarily driven by changes to intrinsic properties, while the excitability shifts following D2R antagonism relied on synaptic transmission. In contrast, chronic depletion of dopamine to the motor circuit with 6-hydroxydopamine induced layer-specific synaptic transmission-dependent shifts in M1 neuron excitability that only partially overlapped with the effects of acute D1R antagonism. These results suggest that while acute and chronic changes in dopamine modulate the input/output function of M1 neurons, the mechanisms engaged are distinct depending on the duration and origin of the manipulation. Our study highlights the broad influence of dopamine on M1 excitability by demonstrating the consequences of local and global dopamine depletion on neuronal input/output function.

## Significance Statement

Dopaminergic signaling is crucial for the control of voluntary movement, and loss of dopaminergic transmission in the motor circuit is thought to underlie motor symptoms in those with Parkinson’s disease (PD). Studies in animal models of PD highlight changes in M1 activity following dopamine depletion; however, the mechanisms underlying this phenomenon remain poorly understood. Here we show that diminished dopamine signaling significantly alters the excitability and input/output function of M1 pyramidal neurons. The effects differed depending on the mode and location—local versus across the motor pathway—of the dopamine manipulation. Our results demonstrate how loss of dopamine can engage complex mechanisms to alter M1 neural activity.

## Introduction

Primary motor cortex (M1) exerts powerful control over movement execution through its central location in the motor circuit. It receives inputs from other cortices and the thalamus, the latter relaying converging signals from the basal ganglia and cerebellum (Mink, 1996; Bosch-Bouju et al., 2013; Hooks et al., 2013), and makes direct connections to descending motor tracts (Lemon, 1993). Pyramidal neurons in each layer of M1 show distinct projection patterns, with neurons in the superficial layers largely innervating deep layers of M1, other cortices, or striatum; neurons in deep layers primarily project back to the thalamus, striatum, or the corticospinal tract (Oswald et al., 2013). Integral to the transition from input to motor output is neural excitability, which influences the magnitude of the response of a neuron to incoming activity. Excitability can be modulated by motor learning and synaptic plasticity, as well as by changes in overall synaptic drive (Paz et al., 2009; Kida et al., 2016). Neuromodulators further contribute to regulating excitability and input/output functions of neurons. The most crucial neuromodulator for the central control of movement is dopamine.

The influence of dopamine on M1 excitability remains poorly understood (Vitrac and Benoit-Marand, 2017). Anatomical studies report that dopaminergic neurons extend projections to M1 (Fallon and Moore, 1978; Williams and Goldman-Rakic, 1998; Hosp et al., 2011), and dopaminergic activity modulates M1 neurons firing rates (Vitrac et al., 2014). Direct dopaminergic projections are most dense in the deep layers of rodent M1 (Nomura et al., 2014), and D1 receptors (D1Rs) and D2Rs are expressed along the entire depth of the cortical column with laminar-specific density (Ariano and Sibley, 1994; Khan et al., 1998; Lemberger et al., 2007), suggesting possible laminar differences in the effects of local dopamine modulation.

However, the largest dopaminergic input to the motor circuit projects from the substantia nigra pars compacta (SNc) to the basal ganglia (Beckstead et al., 1979), where it signals through D1R and D2R with opposing effects on neural excitability (Surmeier et al., 2007; Azdad et al., 2009; Planert et al., 2013). This, along with its control of synaptic plasticity and transmission, make dopamine a powerful regulator of basal ganglia output (Bagetta et al., 2010) with the ability to affect activity across the motor circuit.

Diminished dopaminergic input to the motor circuit can profoundly impair movement, as observed in Parkinson’s disease (PD). While it is clear that depleted dopamine leads to shifts in excitability and synaptic transmission (Blandini et al., 2000; Jankovic, 2008; Grieb et al., 2013; Calabresi and Di Filippo, 2015), this evidence is largely restricted to studies of the basal ganglia. It is unclear if altered basal ganglia activity following nigral dopamine depletion impacts the input/output function of M1 neurons. The classic model of PD postulates that loss of dopamine in the basal ganglia increases the inhibitory output of these nuclei and leads to decreased activation of M1 (Albin et al., 1989; McGregor and Nelson, 2019), which could consequently alter excitability. Furthermore, patients with PD exhibit reduced dopaminergic axon density directly in M1, and functional studies of patients as well as animal models of the disease show altered M1 neural activity, suggesting that depleted dopamine within M1 could also play a role in shifting M1 neural excitability (Gaspar et al., 1991; Lefaucheur, 2005; Lindenbach and Bishop, 2013).

Here, we examined the effect of acute and chronic loss of dopamine signaling on the input/output function of M1 pyramidal neurons using patch-clamp recordings in acute brain slices. First, we tested how acute blockade of D1R and D2R affects neuronal excitability in superficial and deep layers of M1. We then asked whether chronic loss of dopaminergic signaling either in the midbrain or locally within M1 impacts M1 neural excitability. Our results show laminar-specific effects on M1 input/output function through multiple mechanisms. Acute antagonism of D1R and D2R increased excitability through a mix of synaptic transmission dependent and independent mechanisms. Chronic depletion of dopaminergic neurons in the midbrain was also sufficient to engage synapse-dependent modulation of M1 neurons input/output function, although the effects only partially overlapped with those observed following acute blockade. These data show that loss of dopamine impacts M1 neural excitability and highlights the complex mechanisms that can be engaged depending on laminar location within M1 and specific manipulation of dopamine signaling.

## Materials and Methods

### Experimental procedures

Surgical and experimental procedures followed the guidelines of the National Institutes of Health and were approved by the institutional Animal Care and Use Committee. C57BL/6 mice of both sexes were used in the following experiments. The number of animals and the number of cells in each experimental group were shown as *N* and *n*, respectively, in the figure legends.

#### 6-Hydroxydopamine injection

Chronic dopamine depletion was achieved by injection of 6-hydroxydopamine (6OHDA) either in the SNc or in M1. Before surgery, animals received an intraperitoneal injection of desipramine (1.25 mg/ml, 20 ml/kg) to protect noradrenergic and serotonergic afferents from taking up 6OHDA. C57BL/6 mice of both sexes [postnatal day 35 (P35) to P45] were anesthetized (100 mg/kg ketamine and 10 mg/kg xylazine), and a craniotomy was made over the injection site of interest. The 6OHDA solution (15 mg/ml in 0.02% ascorbate) was prepared fresh at the time of injection. Mice unilaterally injected within M1 received 3 μg of 6OHDA or vehicle via two 100 nl injections (bregma, 1.2/0.8 mm, midline, 1.1 mm; surface, 0.8 mm). Animals unilaterally injected in the SNc received 7.5 μg of 6OHDA or vehicle via two 250 nl injections (bregma, 3.1/2.8; midline, 1.2; surface, 3.93). A pressure injection system (Nanoinject, Drummond) was used for these procedures. Following surgery, animals were monitored daily for food and water intake, and administered fluids or softened food when needed.

#### Cylinder motor task

Before slice preparation, all 6OHDA or vehicle-injected animals were assessed for motor impairment using the cylinder motor task (Iancu et al., 2005). The animal was placed in a clear acrylic cylinder and allowed to freely explore for 10 min while being filmed with a camera positioned on top of the cylinder. Mirrors were positioned around the cylinder to facilitate visualization of forelimb use and *post hoc* analysis. Weight-bearing forelimb wall touches were counted over a 3 min period, or a minimum of 20 touches, by an experimenter who was blind to the surgical procedures. Use of the forelimb was quantified as a ratio of wall touches by the forelimb contralateral to the injection (vehicle or 6OHDA) over total wall touches.

#### Slice electrophysiology

Animals at P50 to P70, or 2–3 weeks after surgery for the vehicle- and 6OHDA-injected groups, were deeply anesthetized with isoflurane using the bell jar method and rapidly decapitated. Following dissection of the brain, acute 300 μm slices containing forelimb M1 (Tennant et al., 2011) were prepared using a vibrating blade microtome (model VT1000S, Leica). Tissue was sectioned in ice-cold oxygenated artificial CSF (ACSF), recovered for 30 min in 37°C ACSF, then allowed to stabilize at room temperature for at least 40 min before recording. Whole-cell patch clamp of visually identified excitatory neurons was performed at room temperature using pulled borosilicate glass pipettes with a resistance of 3–4 MΩ. Dynamic input resistance and frequency–current (*f–I*) measurements were obtained in current clamp by injecting 700 ms current steps of increasing intensity (−100 to 450 pA at 50 pA increments). Action potential threshold and half-width were measured on single action potentials at rheobase (rheobase was determined as the current step, in 2 pA increments, that elicited a single action potential). Voltage dependence of the *I*_h_ current was measured in current clamp, as the amplitude of the voltage sag current induced by 700 ms hyperpolarizing current steps from –200 to –25 pA, in 25 pA increments. To block dopamine receptors, either a D1 (SCH23390) or a D2 (sulpiride) receptor antagonist was bath applied for 15 min following a 10 min baseline. To assess the dependency of dopaminergic activity blockade on synaptic transmission, bath application of D1 or D2 receptor antagonists was repeated in the presence of fast synaptic transmission blockers (APV, DNQX, and picrotoxin). Synaptic transmission blockers were circulated for 10 min before the application of the dopamine receptor antagonists, and during this time spontaneous activity was monitored in voltage clamp to ensure that all synaptic events onto the recorded cell were abolished. For experiments including bath application of dopamine to provide a dopamine tone to the slice, recordings were performed in the dark to prevent degradation of dopamine. Recorded neurons were exposed to a dopamine solution for 10 min before baseline excitability was measured. After that, the bath application of a solution containing dopamine, SCH23390, and sulpiride started. Cells were incubated in this cocktail for 15 min before excitability was measured. Series resistance (R_s_) was monitored throughout all experiments, and data from cells with R_s_ > 10% of input resistance or changing >20% throughout the recording were excluded from the analysis.

#### Solutions

ACSF used in all electrophysiology experiments contained the following (in mm): 126 NaCl, 3 KCl, 25 NaHCO_3_, 1 NaHPO_4_, 2 MgSO_4_, 2 CaCl_2_, and 14 dextrose. The internal solution contained the following (in mm): 100 K-Glu, 20 KCl, 10 K-HEPES, 4 Mg-ATP, 0.3 Na-GTP, 10 Na-phosphocreatine, and 0.4% biocytin, pH 7.35, titrated with KOH and adjusted to 295 mOsm with sucrose (E_rev_[Cl^–^] = −49.8 mV). D1 and D2 receptor antagonists SCH23390 and (S)-(–)-sulpiride (Tocris Bioscience) were prepared in DMSO and diluted in ACSF to a final concentration of 10 μm. Solutions containing these antagonists were kept in the dark and bath applied during recording. The baseline ACSF for experiments in which a dopamine tone was provided to the slice contained 10 μm dopamine (dopamine hydrochloride, Sigma-Aldrich), 50 μm sodium metabisulfite (Sigma-Aldrich), and 0.2% DMSO, which was added to the solution to balance the DMSO concentration needed to dissolve D1R and D2R blockers. In these experiments, the D1R and D2R blockers SCH23390 and sulpiride stock solutions in DMSO were added to their final concentration to a fresh, oxygenated volume of the dopamine-ACSF that did not contain DMSO. Experiments performed in the presence of synaptic transmission blockers used ACSF containing the following (in μm): 20 DNQX, 50 AP5, 20 picrotoxin. The maximum concentration of DMSO for any experimental condition was 0.3%. In experiments using dopamine, there was no net increase in DMSO across conditions. In experiments in which synaptic blockers were added prior and during bath application of D1R or D2R antagonist the transition from pre- to post-dopamine receptor antagonism did not exceed an increase of 0.1% DMSO.

#### Immunohistochemistry

Recorded slices, along with the remaining brain tissue, were postfixed in 4% paraformaldehyde (0.01 m PBS) pH 7.4, for a minimum of 1 week. Remaining brain tissue containing injection sites was sectioned in the coronal plane at 50 μm using a vibrating blade microtome (model VT1000S, Leica) and stored in PBS at 4°C. Recorded slices were rinsed in PBS and incubated for 30 min at 45°C in an antigen retrieval solution (10 mm sodium citrate, pH 8.5). Slices were again rinsed in PBS, incubated for 2 h in 50 mm glycine at room temperature (RT), then following an additional rinse, were preblocked for 3 h at RT in PBS containing 5% bovine serum albumin (BSA; Sigma-Aldrich), 5% normal goat serum (NGS; Vector Laboratories), and 1% Triton X (Tx; VWR). Slices were then incubated overnight at 4°C in an antibody stock solution containing PBS, 1% BSA, 1% NGS, 0.1% Tx, and the following antibodies: streptavidin Alexa Fluor 568 (1:2000; catalog #S11226, Thermo Fisher Scientific) and mouse anti-GAD67 (1:500; catalog #MAB5406, EMD Millipore). Slices were then rinsed and incubated for 6 h at RT in the same antibody stock solution, containing goat anti-mouse Alexa Fluor 647 (1:500; catalog #A-21235, Thermo Fisher Scientific), and were counterstained with Hoechst 33342 stain (1:5000; catalog #H3570, Thermo Fisher Scientific). Following a final rinse in 0.1 m phosphate buffer (PB), slices were mounted on gelatin-coated slides and coverslipped with fluorescent mounting medium (Fluoromount-G, Thermo Fisher Scientific).

To assess dopamine neuron or bouton loss, free-floating sections containing injection sites from vehicle or 6OHDA treatments, as well as M1, were rinsed and incubated in antigen retrieval solution and glycine, as described above. Sections were incubated in 0.3% hydrogen peroxide (Thermo Fisher Scientific) for 30 min and rinsed, and endogenous avidin and biotin were blocked (Avidin/Biotin Blocking Kit, Vector Laboratories). Following an additional rinse, sections were preblocked in the previously described solution with 0.2% Tx for 1 h. Sections were then incubated overnight at 4°C in the antibody stock solution with 0.1% Tx and rabbit anti-tyrosine hydroxylase (TH; 1:1000; catalog #ab112, Abcam), rinsed, and incubated for 3 h at 4°C in the antibody solution containing biotinylated goat anti-rabbit (1:200; catalog #BA-1000, Vector Laboratories). Sections were rinsed and incubated in avidin-biotin horseradish peroxidase (Vectastain Elite ABC Kit, Vector Laboratories) for 1 h at RT, rinsed, and developed for 60 s in diaminobenzidine (DAB; Peroxidase Substrate Kit, Vector Laboratories). At the end of this process, sections were rinsed in PB, mounted on gelatin-coated slides, and air dried. Slides were then dehydrated in a series of alcohols (70%, 95%, 100%), cleared in xylenes, and coverslipped with Entellan mounting medium.

For determination of cortical layers, a subset of animals was transcardially perfused first with PBS followed by 4% paraformaldehyde. Brains were dissected and postfixed for 24 h in 4% paraformaldehyde then sectioned as described above. The 50 μm sections containing M1 were stained with the same methods as above for the expression of the cytoarchitectural marker SMI-32 (1:2000; mouse anti-SMI32, catalog #801701, BioLegend; 1:500; goat anti-mouse Alexa Fluor 488, catalog #A-11001, Thermo Fisher Scientific) and counterstained with a pancellular nuclear stain (1:5000; Hoechst 33342 stain; catalog #H3570, Thermo Fisher Scientific) and neuronal-targeting fluorescent Nissl stain (1:200; Neurotrace 530/615, catalog #N21482, Thermo Fisher Scientific). Imaging of fluorescently labeled sections was performed on a laser-scanning confocal microscope (FluoView, Olympus), and bright-field images were obtained using a wide-field microscope (Olympus).

#### Stereological analysis

The effect of vehicle or 6OHDA on dopaminergic neurons in the SNc and ventral tegmental area (VTA), and on putative dopaminergic boutons in M1 were assessed using unbiased stereological methods with the Stereo Investigator System (MBF Bioscience; Gundersen, 1986; Grieb et al., 2013). These assessments were performed by an experimentalist who was blind both to surgical procedures and electrophysiological results. Sections containing injection sites were processed as described for TH, which labels dopaminergic neurons in the midbrain. Contours of the SNc, VTA, and layers in M1 were traced based on cytoarchitectural bounds from Nissl-stained adjacent sections, combined with chemoarchitectonic delineations as previously described (Fu et al., 2012). For counts of TH^+^ neurons in the SNc, eight sections spaced 150 μm apart were counted at 400× magnification, using a grid size of 150 × 150 μm and a 100 × 100 μm counting frame. The dissector height was set to 20 μm with a guard zone of 2 μm. Counts of TH^+^ neurons in the VTA were performed in the same manner, with six sections per animal. TH^+^ axon varicosities (putative boutons) were counted in layer 2/3 and layer 5, across two consecutive 50 μm sections at 1000× magnification, using a grid size of 100 × 100 μm and a 40 × 40 μm counting frame. The dissector height was set to 20 μm with a guard zone of 2 μm. These sampling parameters were sufficient to yield population estimates with a Gunderson coefficient of error of <10% for the unlesioned hemispheres and were applied to all cases used in the study. Lesion severity was expressed as a ratio of the estimated population of TH^+^ neurons, or boutons, in the region of interest of the injected hemisphere, relative to that of the contralateral hemisphere. Animals with lesion quantification falling beyond 2 SDs of the mean were excluded.

### Data analysis

Analysis of electrophysiological data was performed with custom-made procedures in Igor (WaveMetrics). Dynamic input resistance was computed as the slope of the current–voltage curve obtained from a series of hyperpolarizing current steps (−100 to 0 pA). Rheobase was determined by injecting current steps at 2 pA increments until reaching the generation of a single action potential. Action potential threshold and half-width were both calculated at rheobase, in the following manner: (1) the *x*-axis coordinate of the last zero crossing preceding the maximum of the second derivative of the trace was calculated; and (2) this *x* position was then applied to the original trace, and the threshold was determined to be the *y* value at this point. To calculate action potential half-width, the action potential amplitude was calculated as the difference in membrane potential between the peak of the action potential and the threshold, then the half-width was calculated as the duration of the action potential at the voltage halfway between action potential threshold and peak amplitude. *f–I* curves were computed as the average frequency of action potentials for a given current step across all cells in each experimental group (50–450 pA). Voltage dependence of *I*_h_ was measured as the difference in membrane potential between absolute minima of the membrane potential within the first 300 ms of the current step and the average of the steady-state portion of the current step (the last 200 ms) across hyperpolarizing steps.

### Data presentation and statistical analysis

Data were compiled and analyzed in Microsoft Excel and the add-in statistical program XLSTAT. Data obtained from the cylinder motor assessment and stereological counts are presented as the mean ± SEM for the number of animals (*N*) indicated. Voltage dependence on *I*_h_ and *f–I* curve data is represented as line plots where each data point is the mean ± SEM of the pooled number of neurons (*n*) across animals (*N*) indicated in the legend. All other electrophysiology data were imported into https://www.estimationstats.com/ to formulate graphs. Data were tested for normal distribution using the Shapiro–Wilk test. Null hypothesis significance testing between groups was performed using two-tailed unpaired or paired Student’s *t* test or the nonparametric Wilcoxon signed-rank test (for paired data) or Mann–Whitney *U* test (for unpaired data) for data that did not follow a normal distribution (voltage dependence of *I*_h_, *f–I* curves). *p* Values ≤0.05 were considered significant. Where appropriate, we reinforced these analyses using estimation statistics: https://www.estimationstats.com/ was used to import raw data and obtain 95% confidence intervals (CIs) around the mean difference between groups. Bias-corrected and accelerated bootstrap resampling was used to generate 5000 resamples, their distribution, and to construct the 95% CI of the effect size. Individual neurons in drug wash-on experiments are shown as lines as well as the mean ± SEM in modified Cumming estimation plots (Calin-Jageman and Cumming, 2019; Ho et al., 2019). Individual neurons in lesion experiments are shown as swarm plots and the mean ± SEM in modified Cumming estimation plots. Alongside the individual neuron data are black dots showing the mean difference between groups (effect size), vertical bars displaying the 95% CIs, and the underlying resample distribution. Permutation tests were used to determine the likelihood of observing the calculated effect size if the null hypothesis of zero difference was true, and *p* ≤ 0.05 was considered significant. The *p* values for all of the experimental conditions and statistical analyses are reported in Table 1 Layer 2/3 (or L2/3) and Table 2 (L5).

**Table 1:** Summary statistics table for L2/3 neurons across all experiments

Experiment	Parameter	*n*	Mean difference	CI lower limit	CI upper limit	*p* Value	
						Permutation *t* test	Student’s *t* test
ACSF D1ant	DIR	12	29.15	13.25	46.09	**0.010**	**0.007**
	AP threshold	12	–1.86	–2.89	–0.83	**0.009**	**0.007**
	AP half-width	12	0.11	0.05	0.21	**0.011**	**0.017**
BLK D1ant	DIR	12	62.99	48.57	95.31	**0.001**	**0.000**
	AP threshold	10	–1.90	–3.69	–0.58	**0.049**	**0.048**
	AP half-width	10	0.07	0.02	0.12	**0.024**	**0.024**
ACSF D2ant	DIR	11	20.22	8.05	35.17	**0.016**	**0.019**
	AP threshold	11	–1.50	–2.24	–0.61	**0.007**	**0.006**
	AP half-width	11	–0.01	–0.04	0.03	0.476	0.472
BLK D2ant	DIR	8	10.84	1.70	25.40	0.152	0.126
	AP threshold	8	–0.89	–2.62	1.16	0.392	0.403
	AP half-width	8	–0.03	–0.06	0.00	0.125	0.113
DOPA D1+D2ant	DIR	11	26.52	8.57	54.42	**0.032**	**0.046**
	AP threshold	11	–1.99	–2.69	–1.47	**0.000**	**0.000**
	AP half-width	11	0.04	0.01	0.07	**0.050**	**0.042**
SNc 6OHDA ACSF		Vehicle = 16					
	DIR	6OHDA = 21	–9.64	–46.15	28.89	0.595	0.609
		Vehicle = 16					
	AP threshold	6OHDA = 21	0.68	–1.36	2.49	0.501	0.504
		Vehicle = 16					
	AP half-width	6OHDA = 21	0.07	–0.05	0.20	0.305	0.303
SNc 6OHDA BLK		Vehicle = 7					
	DIR	6OHDA = 8	4.78	–82.51	83.74	0.907	0.917
		Vehicle = 7					
	AP threshold	6OHDA = 7	1.50	–2.33	5.91	0.534	0.527
		Vehicle = 7					
	AP half-width	6OHDA = 7	0.13	–0.07	0.35	0.310	0.302
M1 6OHDA BLK		Vehicle = 12					
	DIR	6OHDA = 14	2.90	–31.25	41.27	0.877	0.884
		Vehicle = 12					
		6OHDA = 12	2.35	–0.12	4.53	0.063	0.067
		Vehicle = 12					
	AP half-width	6OHDA = 12	–0.18	–0.33	–0.01	**0.053**	**0.050**

Permutation *t* test *p* values are listed alongside Student’s *t* test *p* values for comparison. Bold *p* values indicate a *p* value ≤ 0.05. AP, Action potential; DIR, dynamic input resistance.

**Table 2:** Summary statistics table for L5 neurons across all experiments

Experiment	Parameter	*n*	Mean difference	CI lower limit	CI upper limit	*p* Value	
						Permutation *t* test	Student’s *t* test
ACSF D1ant	DIR	11	16.95	8.42	27.31	**0.002**	**0.007**
	AP threshold	11	–1.30	–2.10	–0.58	**0.007**	**0.009**
	AP half-width	11	0.02	0.00	0.04	0.067	0.065
BLK D1ant	DIR	12	45.56	30.50	65.71	**0.000**	**0.000**
	AP threshold	10	–1.66	–3.06	–0.38	0.058**	**0.047**
	AP half-width	10	0.03	0.00	0.06	0.092	0.099
ACSF D2ant	DIR	10	9.91	4.09	22.43	0.023**	**0.053**
	AP threshold	10	–2.59	–3.76	–1.40	**0.003**	**0.003**
	AP half-width	10	–0.04	–0.06	–0.02	**0.013**	**0.018**
BLK D2ant	DIR	8	16.94	9.55	36.71	**0.000**	**0.027**
	AP threshold	8	–2.24	–4.83	–0.95	0.024**	0.055
	AP half-width	8	–0.06	–0.08	–0.04	**0.000**	**0.000**
DOPA D1+D2ant	DIR	10	11.08	2.36	20.04	**0.042**	**0.047**
	AP threshold	10	–1.21	–1.71	–0.69	**0.004**	**0.002**
	AP half-width	10	0.04	0.01	0.06	**0.017**	**0.017**
SNc 6OHDA ACSF		Vehicle = 29					
	DIR	6OHDA = 29	–2.55	–23.05	15.48	0.808	0.803
		Vehicle = 29					
	AP threshold	6OHDA = 28	0.67	–0.81	2.15	0.391	0.392
		Vehicle = 29					
	AP half-width	6OHDA = 28	0.08	–0.03	0.19	0.177	0.173
SNc 6OHDA BLK		Vehicle = 14					
	DIR	6OHDA = 12	–26.11	–71.51	–1.13	0.165	0.168
		Vehicle = 14					
	AP threshold	6OHDA = 11	0.05	–2.25	2.56	0.970	0.971
		Vehicle = 14					
	AP half-width	6OHDA = 11	–0.06	–0.19	0.05	0.391	0.396
M1 6OHDA BLK		Vehicle = 16					
	DIR	6OHDA = 16	–9.21	–37.70	16.81	0.532	0.519
		Vehicle = 16					
	AP threshold	6OHDA = 16	0.64	–1.37	2.70	0.547	0.542
		Vehicle = 16					
	AP half-width	6OHDA = 16	–0.06	–0.20	0.07	0.434	0.416

Permutation *t* test *p* values are listed alongside Student’s *t* test *p* values for comparison. Bold *p* values indicate *p* ≤ 0.05. AP, Action potential; DIR, dynamic input resistance.

**Instances where one statistics test was over/under the 0.05 *p* value threshold when the other was not.

## Results

**Figure 1.**
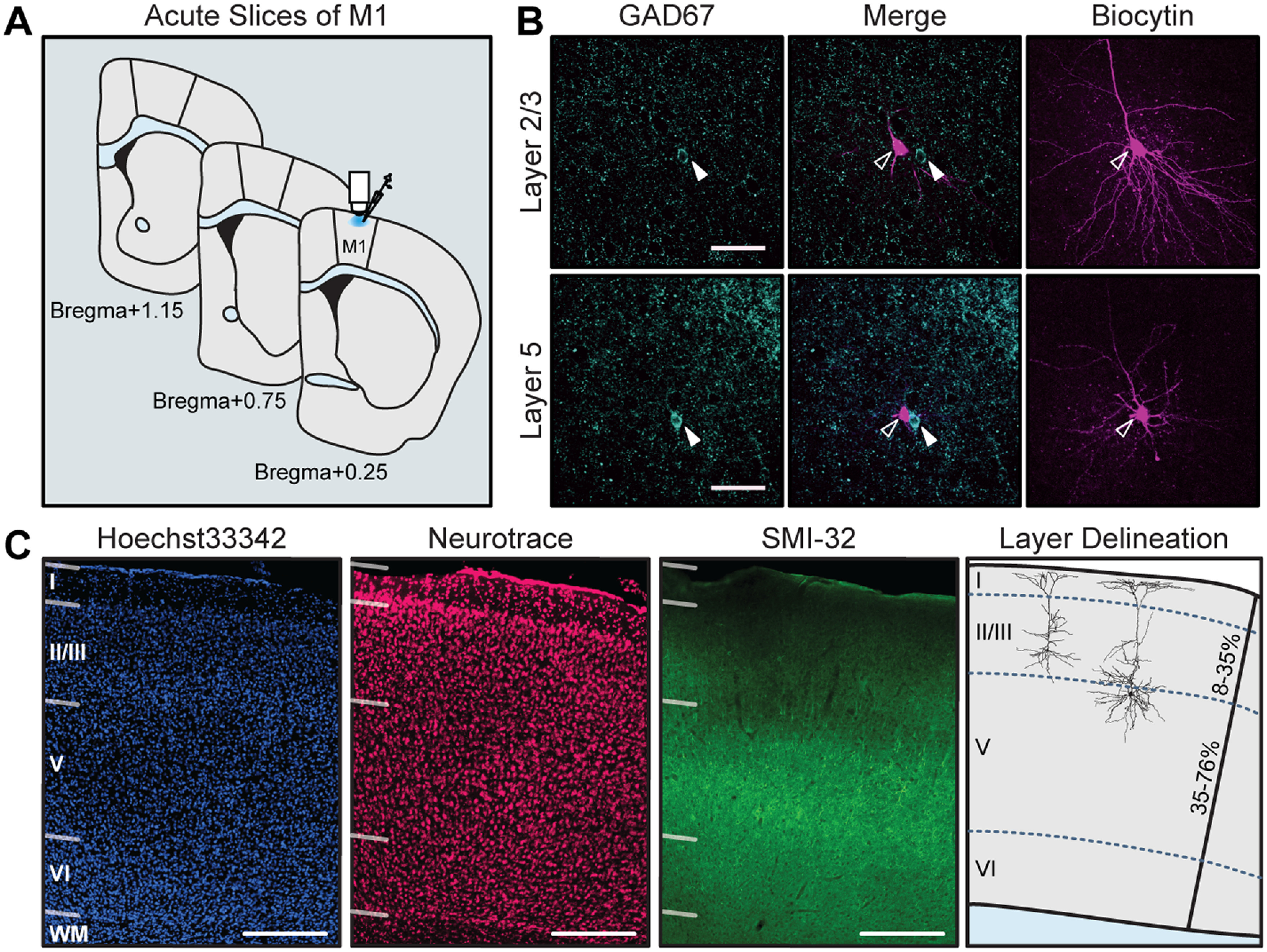
Whole-cell recordings of excitatory neurons in forelimb M1 were localized to L2/3 and L5. ***A***, Schematic showing the anterior–posterior span of recorded slices, restricted to the forelimb area of M1. ***B***, Recorded neurons were visualized with streptavidin labeling of biocytin and confirmed as excitatory by negative immunoreactivity for GAD67. GAD67 and merged images are shown at one *z*-plane depth; biocytin images are shown as a collapsed stack spanning the entire neuron. Open arrows, GAD67^–^ biocytin-filled neurons; closed arrows, neighboring GAD67^+^ interneurons (not recorded) at the same depth. Scale bar, 50 μm. ***C***, Histologic staining of cytoarchitecture used to define cortical layers. Hoechst 33342 stain is a nuclear counterstain of all cells in the region, Neurotrace was used as a neuron-specific stain for somata, SMI-32 labels a subset of pyramidal neurons in layer 3 and layer 5. Scale bar, 200 μm. Right-most panel, Two example neurons localized to L2/3 and L5; neurons localized within 8–35% of the total cortical depth were defined as L2/3; neurons within 35–76% of cortical depth were defined as L5.

We performed whole-cell recordings of excitatory neurons in the superficial and deep layers of the forelimb region of M1 (Fig. 1*A*) to assess the effect of impaired dopamine signaling locally and/or across the motor circuit on neuronal input/output functions. Recorded neurons included in this study showed pyramidal morphology and were negative for GAD67 immunoreactivity (Fig. 1*B*). To determine laminar borders, sections containing M1 were stained for the cytoarchitectural marker SMI-32, a nuclear counterstain (Hoechst 33342 stain), and a fluorescent Nissl (Fig. 1*C*). The top border of Layer 2/3 (L2/3) was placed where cell density sharply drops off as you move toward the pial surface. The bottom of L2/3 was defined at the depth where the cortex transitions from small, densely packed neurons to very large, sparser pyramidal neurons, as visualized with Nissl staining. SMI-32 labels a subset of pyramidal neurons in L2/3 and L5 (Campbell and Morrison, 1989; Voelker et al., 2004), and in dysgranular and agranular cortex it is expressed most strongly in L5 (Barbas and García-Cabezas, 2016). Staining for SMI-32 was used to confirm the previously defined laminar borders, and to demarcate the end of L5 and the beginning of L6. Cells were localized to L2/3 or L5 by determining their depth from the cortical surface with *post hoc* immunostaining of recorded neurons.

### Acute blockade of dopamine receptors increases M1 neural excitability

D1R and D2R in the rodent brain are expressed in neurons across the cortical mantle, although their expressions show laminar preference (Lemberger et al., 2007; Santana et al., 2009). While dopaminergic modulation through these receptors influences activity in M1 (Molina-Luna et al., 2009; Hosp et al., 2011), the mechanisms underlying the regulation of M1 neurons by dopamine are not clear. To assess how D1R and D2R modulate the input/output function of M1 neurons, we first studied the effects of acute dopamine receptor blockade using pharmacological antagonists. We compared current-clamp responses to subthreshold and suprathreshold current steps before and after bath application of dopamine receptor antagonists. To determine whether the modulation of pyramidal neuron input/output function by dopamine receptors is because of intrinsic conductance or synaptic activity, we compared the effect of dopamine receptor antagonists in ACSF, in which spontaneous synaptic activity is present, and in the presence of ionotropic GABA and glutamate receptor blockers.

**Figure 2.**
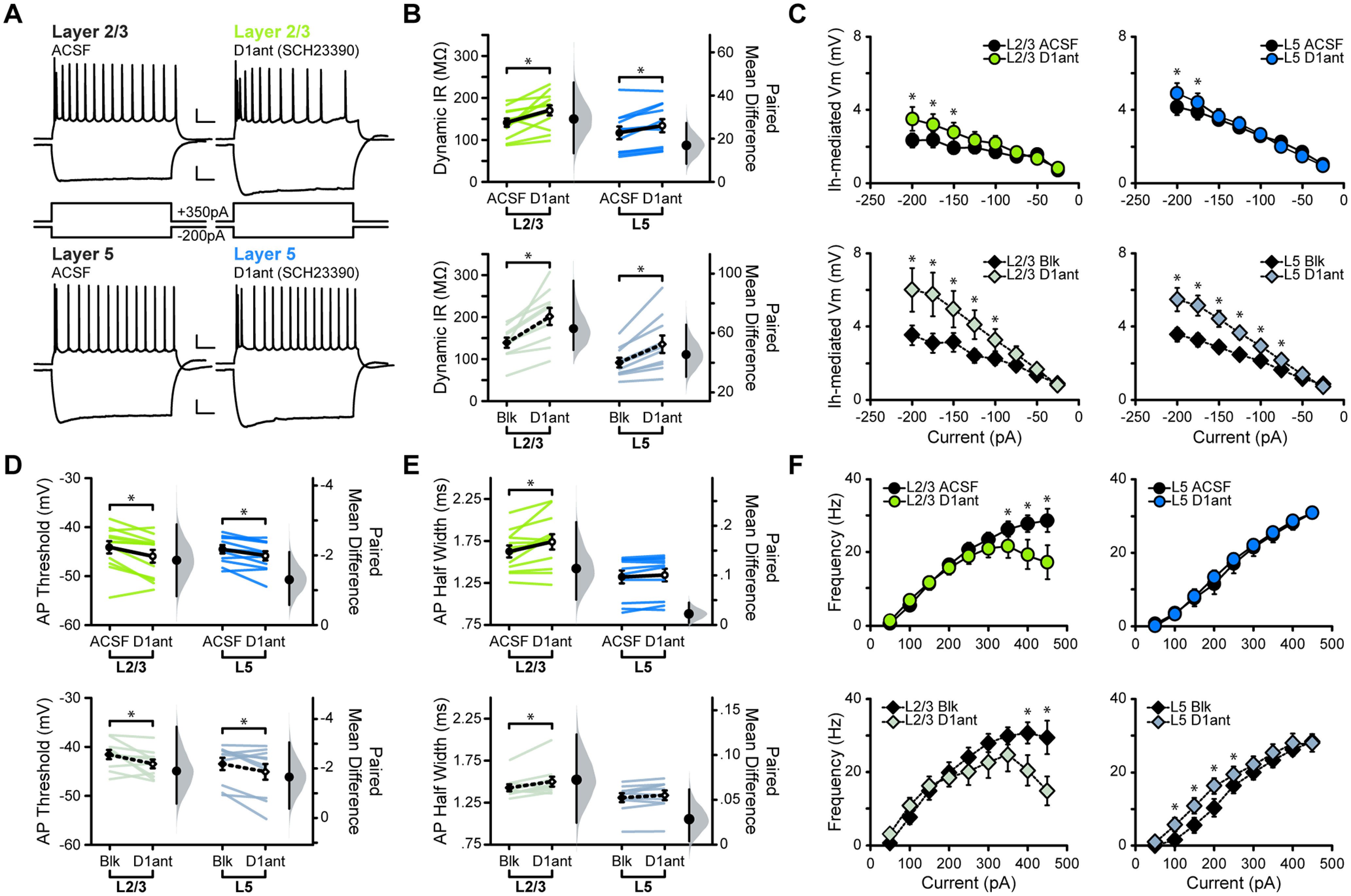
Acute D1R blockade shifts excitability of M1 neurons. ***A***, Superimposed responses to hyperpolarizing and depolarizing current steps in individual L2/3 and L5 neurons before and after bath application of D1R antagonist SCH23390 (D1ant, 10 μm). Scale bar: Top, 20 mV, 100 ms; bottom: 10 mV, 100 ms. ***B–F***, Summary excitability plots for excitatory neurons in L2/3 (green) and L5 (blue) before and after D1ant application, in baseline (ACSF) or synaptic blocker (BLK: 20 μm picrotoxin, 20 μm DNQX, 50 μm AP5) conditions. Modified Cumming plots show raw data of individual neurons as lines, overlayed with the mean ± SEM. To the right of each group of raw data are the effect size (black circle), corresponding 95% CIs (black vertical bars), and the underlying bootstrap sampling distribution. ***B***, Dynamic input resistance across hyperpolarizing current steps. ***C***, Voltage dependence of *I*_h_-mediated voltage sag elicited by hyperpolarizing current. ***D***, Action potential threshold at rheobase. ***E***, Action potential half-width at rheobase. ***F***, Action potential frequency during suprathreshold current injections (ACSF L2/3 neurons: *N* = 6, *n* = 12; ACSF L5 neurons: *N* = 6, *n* = 11; BLK L2/3 neurons: *N* = 5, *n* = 10; BLK L5 neurons: *N* = 6, *n* = 10. Data are shown as the mean ± SEM. *p ≤ 0.05.

In a first set of experiments, we examined the effects of the D1R antagonist (D1ant) SCH23390 (10 μm) on the excitability (for detailed description of analysis, see Materials and Methods) of pyramidal neurons in L2/3 and L5 of M1 (Fig. 2). In ACSF, bath application of SCH23390 increased the dynamic input resistance of both L2/3 and L5 neurons [Fig. 2*B*; in MΩ: L2/3 ACSF, 141.32 ± 9.97; L2/3 D1ant, 170.49 ± 12.24 (*p* = 0.007); L5 ACSF, 106.33 ± 14.80; L5 D1ant, 123.28 ± 15.66 (*p* = 0.007)]. This effect was potentiated in the presence of ionotropic GABA and glutamate receptor blockers (BLK), suggesting that increased dynamic input resistance induced by D1R blockade occurs independent of fast synaptic transmission [Fig. 2*B*; in MΩ: L2/3 BLK, 139.06 ± 12.04; L2/3 D1ant, 201.73 ± 20.41 (*p* = 0.001); L5 BLK, 91.79 ± 11.29; L5 D1ant, 136.25 ± 20.90 (*p* = 0.002)].

In response to hyperpolarizing current steps, neurons showed a voltage sag during the initial portion of the response, which is typically associated with the presence of hyperpolarization-activated cation channels (HCNs) mediating *I*_h_ (Rosenkranz and Johnston, 2006; Hogan and Poroli, 2008). Bath application of SCH23390 increased the amplitude of the voltage sag in both L2/3 and L5 neurons, and this change persisted in the presence of synaptic blockers, confirming the independence of this effect from fast synaptic transmission (Fig. 2*C*).

Next, we assess the effect of D1R blockade on the suprathreshold portion of the input/output function. We first compared action potential properties at rheobase: the action potential threshold was more hyperpolarized in both L2/3 and L5 neurons in the presence of SCH23390 [Fig. 2*D*; in mV: L2/3 ACSF, −44.10 ± 1.26; L2/3 D1ant, −45.96 ± 1.28 (*p* = 0.0073); L5 ACSF, −44.57 ± 0.84; L5 D1ant, −45.87 ± 0.93 (*p* = 0.0087)]. When this experiment was repeated in the presence of fast synaptic transmission blockers, the effect remained in both layers [Fig. 2*D*; in mV: L2/3 BLK, −41.57 ± 0.93; L2/3 D1ant, −43.47 ± 0.88 (*p* = 0.048); L5 BLK, −43.49 ± 1.25; L5 D1ant, −45.14 ± 1.63 (*p* = 0.047)]. Blocking D1Rs also increased action potential half-width in L2/3, but not in L5 neurons [Fig. 2*E*; in ms: L2/3 ACSF, 1.63 ± 0.071; L2/3 D1ant, 1.74 ± 0.091 (*p* = 0.017); L5 ACSF, 1.32 ± 0.077; L5 D1ant, 1.35 ± 0.075 (*p* = 0.065)]. This effect of D1R blockade on L2/3 neuron half-width persisted in synaptic transmission blockers [Fig. 2*E*; in ms: L2/3 BLK, 1.43 ± 0.041; L2/3 D1ant, 1.50 ± 0.059 (*p* = 0.024); L5 BLK, 1.31 ± 0.053; L5 D1ant, 1.34 ± 0.058 (*p* = 0.099)]. Thus, dopamine affects action potential properties of M1 pyramidal neurons by acting through distinct mechanisms in superficial and deep layers.

Bath application of the D1R antagonist SCH23390 unveiled laminar differences in the effects of acute D1R blockade on the input/output curve. Comparison of relationships between action potential frequency and injected current (*f–I* curve) in ACSF and acute D1R blockade showed that the ability of L2/3 neurons to increase their firing rates in response to increasing current steps was impaired, significantly reducing the maximum firing rate. In contrast, SCH23390 did not affect the *f–I* curve of L5 neurons (Fig. 2*F*). The changes in the *f–I* curve of L2/3 neurons persisted in synaptic blockers, indicating that they depend on the modulation of voltage-gated conductance. Interestingly, in L5, pharmacological blockade of ionotropic synaptic receptors unveiled a previously masked effect of D1R blockade on the *f–I* curve: an increase in firing rate selectively in the linear portion of the *f–I* curve, the range in which firing rates of neurons show high sensitivity to small changes in current injection. The increase in input resistance and hyperpolarization of action potential threshold points to a net increase in M1 pyramidal neuron excitability in the absence of dopamine activation of D1R. In L2/3, selectively blocking dopamine signaling through D1R also results in a decreased maximum firing rate, suggesting that large incoming input would result in reduced output.

**Figure 3.**
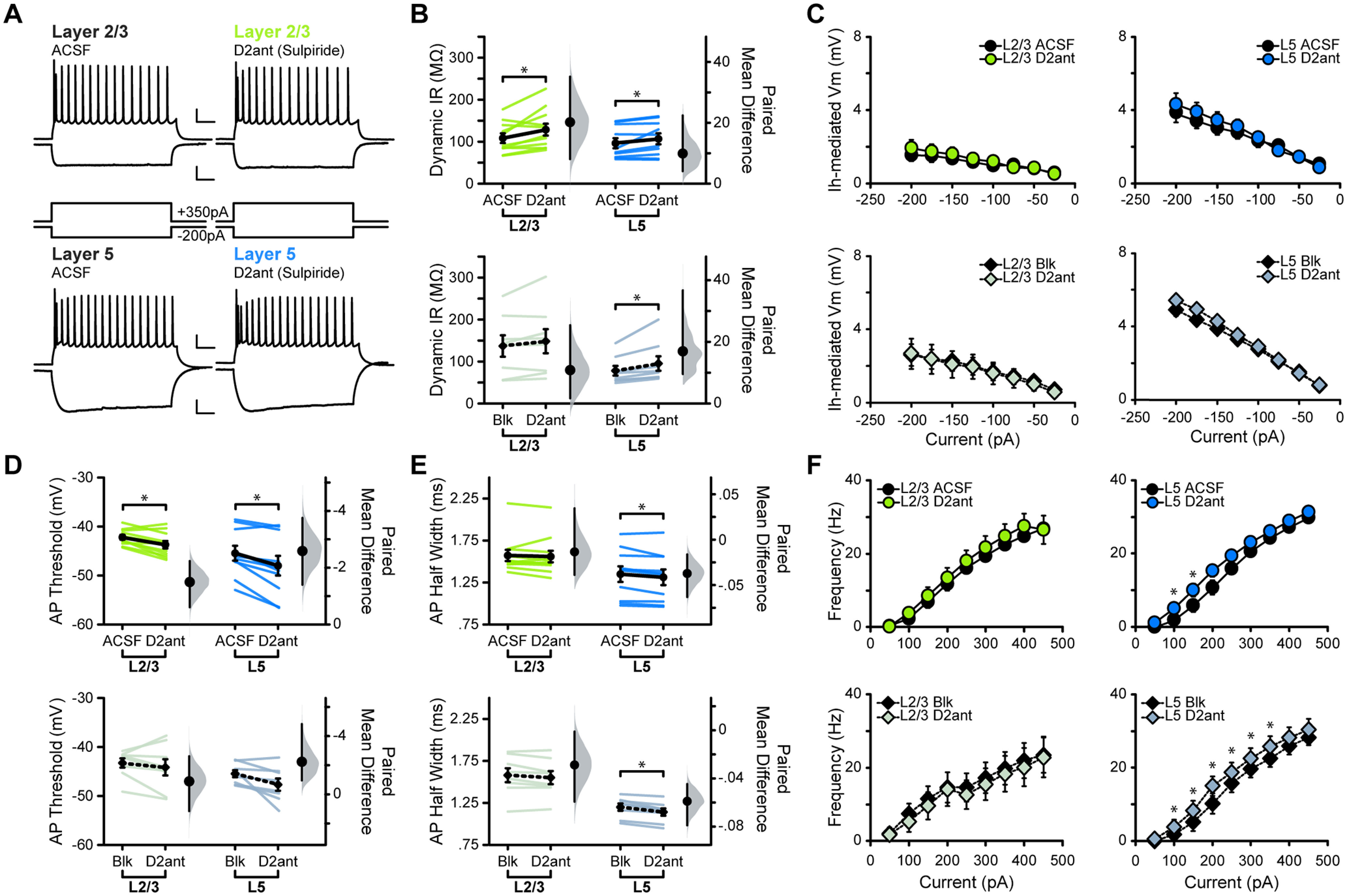
Acute D2R blockade shifts excitability of M1 neurons. ***A***, Superimposed responses to hyperpolarizing and depolarizing current steps in individual L2/3 (green) and L5 (blue) neurons before and after bath application of D2R antagonist sulpiride (D2ant, 10 μm) Scale bar: Top, 20 mV, 100 ms; bottom, 10 mV, 100 ms. ***B–F***, Summary excitability plots for excitatory neurons in L2/3 and L5 before and after D2ant application, in baseline (ACSF) or synaptic blocker (BLK: 20 μm picrotoxin, 20 μm DNQX, 50 μm AP5) conditions. Modified Cumming plots show raw data of individual neurons as lines, overlayed with the mean ± SEM. To the right of each group of raw data are the effect size (black circle), corresponding 95% CIs (black vertical bars), and the underlying bootstrap sampling distribution. ***B***, Dynamic input resistance across hyperpolarizing current steps. ***C***, Voltage dependence of *I*_h_-mediated voltage sag elicited by hyperpolarizing current. ***D***, Action potential threshold at rheobase. ***E***, Action potential half-width at rheobase. ***F***, Action potential frequency during suprathreshold current injections. ACSF L2/3 neurons: *N* = 5, *n* = 11; ACSF L5 neurons: *N* = 5, *n* = 10; Blk L2/3 neurons: *N* = 4, *n* = 8; Blk L5 neurons: *N* = 4, *n* = 8. Data are shown as mean ± SEM. *p ≤ 0.05.

Dopaminergic modulation of neuronal activity can also rely on D2Rs, which are expressed in M1 neurons (Santana et al., 2009). Previous *in vivo* studies reported that the activation of D2Rs increases the firing rate of M1 pyramidal neurons in anesthetized animals and can alter motor maps, but the mechanisms underlying these effects are unclear (Hosp et al., 2009; Vitrac et al., 2014). To assess whether D2Rs modulate the input/output curve of M1 pyramidal neurons, we repeated the experiments above using the D2R antagonist (D2ant) sulpiride (10 μm; Fig. 3). Bath application of sulpiride increased the input resistance of L2/3 and L5 neurons [Fig. 3*B*; in MΩ: L2/3 ACSF, 108.56 ± 11.01; L2/3 D2ant, 127.78 ± 14.12 (*p* = 0.019); L5 ACSF, 97.1763 ± 11.94; L5 D2ant, 107.09 ± 12.69 (*p* = 0.053)]. The increase in input in L2/3 neurons was eliminated by GABA_A_, AMPA, and NMDA receptor antagonists, suggesting that it relies on the modulation of synaptic transmission. In contrast, the application of sulpiride in L5 in the presence of synaptic receptor blockers amplified the increase in input resistance [Fig. 3*B*; in MΩ: L2/3 BLK, 137.33 ± 25.23; L2/3 D2ant, 148.17 ± 28.67 (*p* = 0.13); L5 BLK, 78.69 ± 11.69; L5 D2ant, 95.63 ± 17.39 (*p* = 0.027)]. Sulpiride did not affect the voltage sag in either L2/3 or L5 pyramidal neurons (Fig. 3*C*), suggesting that D2Rs do not modulate this current in M1.

Bath application of sulpiride hyperpolarized the action potential threshold [Fig. 3*D*; in mV: L2/3 ACSF, −42.22 ± 0.49; L2/3 D2ant, −43.72 ± 0.75 (*p* = 0.0063); L5 ACSF, −45.46 ± 1.51; L5 D2ant, −48.05 ± 2.00 (*p* = 0.003)]. In both layers, this effect was eliminated by the presence of ionotropic GABA and glutamate receptor blockers [Fig. 3*D*; in mV: L2/3 BLK, −43.26 ± 0.98; L2/3 D2ant, −44.15 ± 1.68 (*p* = 0.41); L5 BLK, −45.48 ± 0.75; L5 D2ant, −47.72 ± 1.25 (*p* = 0.055)]. Sulpiride had no effect on action potential half-width of L2/3 neurons, but it did decrease the half-width of L5 cells in both ACSF and synaptic blockers [Fig. 3*E*; in ms: L2/3 ACSF, 1.57 ± 0.067; L2/3 D2ant, 1.56 ± 0.069 (*p* = 0.47); L5 ACSF, 1.35 ± 0.093; L5 D2ant, 1.31 ± 0.093 (*p* = 0.018); L2/3 BLK, 1.58 ± 0.083; L2/3 D2ant, 1.55 ± 0.078 (*p* = 0.11); L5 BLK, 1.20 ± 0.043; L5 D2ant, 1.14 ± 0.043 (*p* = 4.28 × 10^−4^)]. This effect in L5 opposes that of D1R antagonism, suggesting that D2R antagonism plays a unique role in modulating action potential properties in M1. Finally, sulpiride had no effect on the *f–I* curve of L2/3 pyramidal neurons. However, in L5 it increased the action potential frequency in the linear portion of the *f–I* curve, increasing L5 neuron output to small changes in input current. The effect of sulpiride on the *f–I* curve of L5 neurons persisted in the presence of synaptic blockers, suggesting that this effect is independent of fast synaptic transmission (Fig. 3*F*). Taken together, these data suggest that while D2Rs play a role in M1 excitability, the consequences of an acute loss of D2R activity are more subtle than those of D1R antagonism. In both layers, acute blockade of D2R signaling results in a net increase in excitability. While in L2/3 this effect depends on synaptic transmission, in L5 it is primarily dependent on the modulation of intrinsic conductance.

To provide additional statistical analysis of these results, we performed estimation statistics on input resistance, threshold, and half-width data from dopamine receptor antagonism experiments. These analyses measured effect size (mean difference) between control and test groups and use bootstrap resampling to construct 95% CIs for each parameter. Permutation *t* tests were used to assess the likelihood of observing the effect size if the null hypothesis of zero difference were true. Permutation *t* test *p* values were consistent with the reported Student’s *t* test values in nearly all instances (Tables 1 for L2/3 neurons, 2 for L5 neurons). In the three cases where the permutation test results differed in significance from the Student’s *t* test (L5; Table 2, marked with asterisks), the *p* values hovered around 0.05. Consistency in both statistical analyses reinforces a physiological role of dopamine receptor activity in M1 and strengthens the assertion that loss of D1 or D2 receptor function impacts the excitability of neurons in L2/3 and L5. These results point to the laminar specificity of dopamine receptor function in M1 and support the hypothesis that dopamine modulates M1 excitability through complex mechanisms involving both intrinsic conductance and synaptic transmission.

### Effects of D1R and D2R antagonism persist in the presence of dopamine tone

Our data indicate that dopaminergic modulation plays a role in maintaining excitability of pyramidal neurons in M1. One consideration when interpreting these results is that while *ex vivo* brain slices preserve much of the synaptic circuitry, endogenous levels of dopamine in M1 may be significantly reduced, and dopamine is highly sensitive to oxidation. Furthermore, both SCH23390 and sulpiride were dissolved into a DMSO vehicle, which has been shown to impact excitability after long slice incubations (Tamagnini et al., 2014). To address these factors, we primed acute M1 slices with a dopamine–ACSF solution before bath applying dopamine receptor antagonists. In this experiment, the concentration of DMSO remained constant before and after the application of dopamine receptor blockers. Considering that previous results showed that D1R and D2R antagonism drove partially overlapping shifts in M1 neuron excitability, we combined SCH23390 and sulpiride in the antagonist condition and posited that simultaneous D1R and D2R antagonism would yield results similar to those observed with individual antagonists.

**Figure 4.**
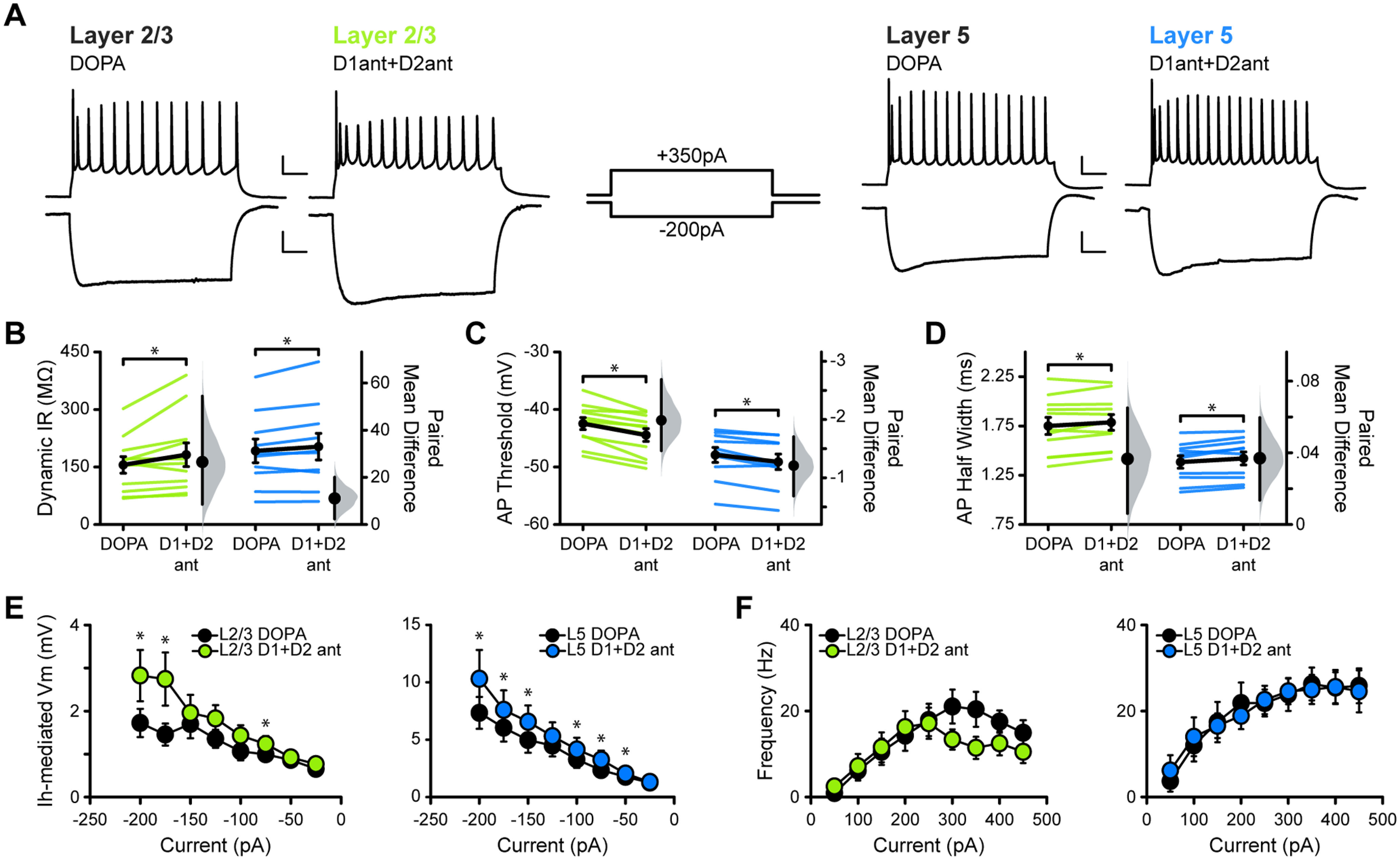
Combined D1R+D2R blockade in dopamine-primed slices of M1 recapitulates the results of individual antagonist experiments. ***A***, Superimposed responses to hyperpolarizing and depolarizing current steps in individual L2/3 (green) and L5 (blue) neurons before and after bath application of SCH23390 (D1ant) and Sulpiride (D2ant). Scale bar: Top, 20 mV, 100 ms; bottom, 10 mV, 100 ms. ***B–F***, Summary excitability plots for excitatory neurons in L2/3 and L5 before and after D1ant+D2ant application, in dopamine (10 μm) ACSF. Modified Cumming plots show raw data of individual neurons as lines, overlayed with the mean ± SEM. To the right of each group of raw data are the effect size (black circle), corresponding 95% CIs (black vertical bars), and the underlying bootstrap sampling distribution. ***B***, Dynamic input resistance across hyperpolarizing current steps. ***C***, Action potential threshold at rheobase. ***D***, Action potential half-width at rheobase. ***E***, Voltage dependence of *I*_h_-mediated voltage sag elicited by hyperpolarizing current. ***F***, Action potential frequency during suprathreshold current injections. DOPA L2/3 neurons: *N* = 7, *n* = 11; DOPA L5 neurons: *N* = 5, *n* = 10. Data are shown as the mean ± SEM. *p ≤ 0.05.

Bath application of the combined antagonists, in the presence of dopamine, reproduced our previous results and reinforced the consequence of reduced dopamine signaling on excitability in M1 (Fig. 4). In both L2/3 and L5 neurons, input resistance was increased [Fig. 4*B*; in MΩ: L2/3 DOPA, 155.62 ± 21.54; L2/3 DOPA+D1ant+D2ant, 182.14 ± 30.86 (*p* = 0.046); L5 DOPA, 192.12 ± 30.76; L5 DOPA+D1ant+D2ant, 203.20 ± 34.73 (*p* = 0.047)], action potential threshold was hyperpolarized [Fig. 4*C*; in mV: L2/3 DOPA, −42.46 ± 1.04; L2/3 DOPA+D1ant+D2ant, −44.45 ± 1.11 (*p* = 0.00011); L5 DOPA, −47.89 ± 1.30; L5 DOPA+D1ant+D2ant, −49.10 ± 1.35 (*p* = 0.002)], and action potential half-width was increased [Fig. 4*D*; in ms: L2/3 DOPA, 1.75 ± 0.086; L2/3 DOPA+D1ant+D2ant, 1.79 ± 0.080 (*p* = 0.042); L5 DOPA, 1.38 ± 0.063; L5 DOPA+D1ant+D2ant, 1.42 ± 0.066 (*p* = 0.017)]. Similar to D1R antagonism alone, combined D1R/D2R antagonism in the presence of dopamine significantly increased the voltage sag in both L2/3 and L5 neurons (Fig. 4*E*). However, there was no significant effect on the *f–I* curve of either L2/3 or L5 neurons, suggesting that simultaneous antagonism of both D1R and D2R may exert a unique effect on the firing rates of neurons in M1 compared with when only one type is blocked (Fig. 4*F*). Analyses of these data using an estimation statistics approach bolstered these results: in all cases, permutation *t* tests of the bootstrap resamples indicate that an acute blockade of D1R and D2R, in the presence of dopamine, drives an increase in the excitability of L2/3 and L5 neurons in M1 (Tables 1, L2/3, 2, L5).

### Chronic midbrain dopamine depletion alters M1 neural excitability

**Figure 5.**
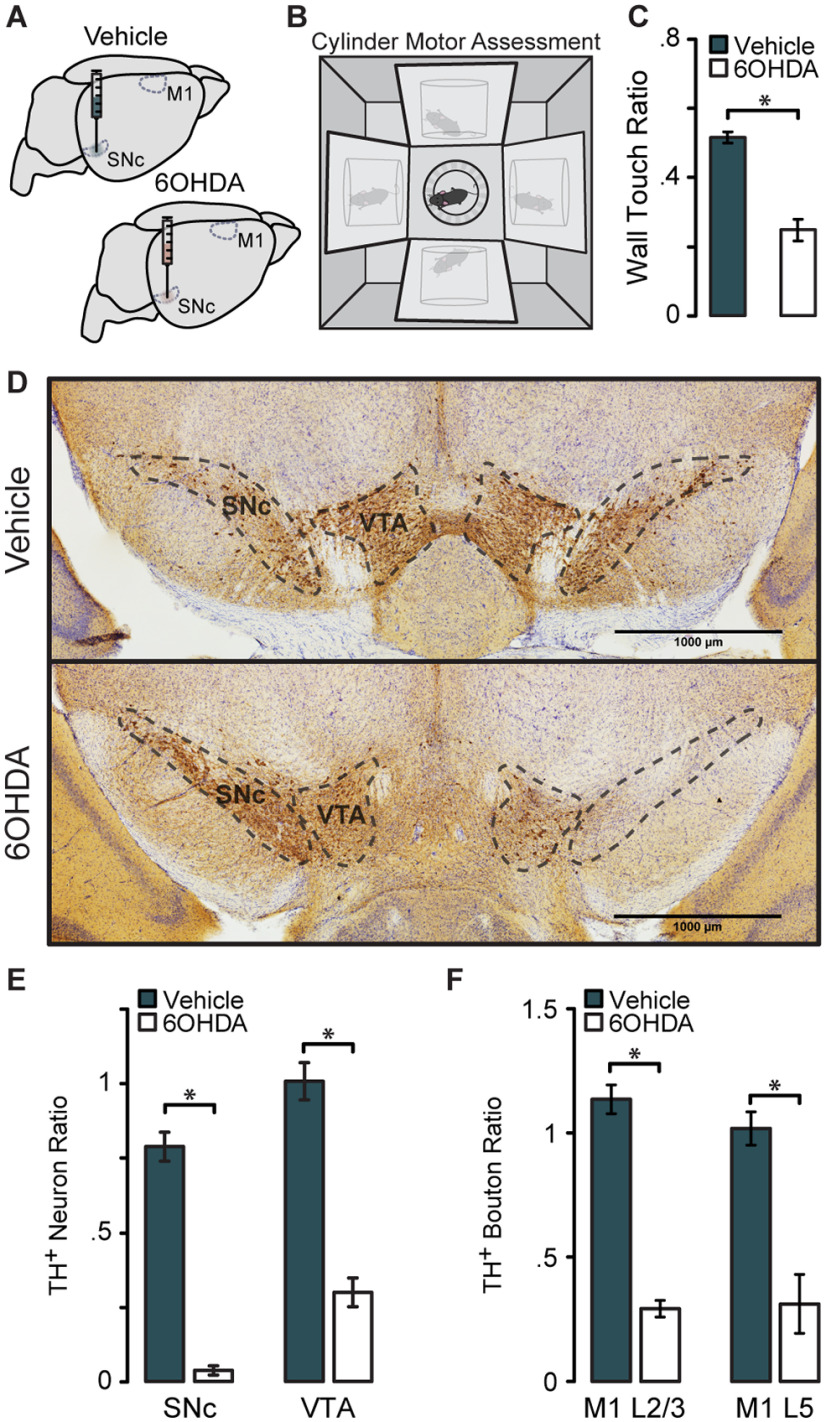
Validation of the 6OHDA model of Parkinson’s disease. ***A***, Unilateral injection of 6OHDA or vehicle, centered on the SNc. ***B***, Schematic of the cylinder motor assessment. ***C***, Quantification of weight-bearing wall touches measured as a ratio of forelimb use contralateral versus ipsilateral to the injected hemisphere. ***D***, Immunolabeled TH^+^ dopaminergic neurons visualized with DAB in the SNc and VTA. ***E***, Summary of stereological counts of TH^+^ neurons in the SNc and VTA of lesioned or vehicle-injected animals. (vehicle animals, *N* = 18; 6OHDA animals, *N* = 17; data are shown as the mean ± SEM. *p < 0.0001. ***F***, Summary stereological counts of TH^+^ boutons in M1 of a subset of animals. Vehicle animals, *N* = 2; 6OHDA animals, *N* = 2. Data are shown as the mean ± SEM. *p ≤ 0.05.

Diminished dopamine signaling in the motor system and progressive motor impairment are hallmarks of PD. Patients, as well as animal models of the disease, exhibit motor cortex dysfunction. We hypothesized that chronic loss of dopaminergic input to the motor circuit alters the excitability of M1 pyramidal neurons, possibly providing a mechanism for impaired motor cortex activity. We asked whether chronic loss of dopaminergic activity across the entire motor circuit, or locally within M1, is sufficient to shift the excitability of M1 neurons, and recapitulate the results of the acute dopamine receptor blockade experiments. We unilaterally injected 6OHDA or an equivalent volume of vehicle as a control into the midbrain centering the injection site on the SNc (Fig. 5). Dopamine depletion of midbrain dopaminergic neurons with 6OHDA is widely used as a model of PD and is known to induce motor impairment. Two weeks after injection, and just before recording, the movement of each animal was assessed with a cylinder motor task (Fig. 5*C*). 6OHDA-injected mice showed reduced use of the forelimb contralateral to the injection, while vehicle-injected animals showed no sign of forelimb use impairment (Fig. 5*C*; vehicle, 0.52 ± 0.017; 6OHDA, 0.25 ± 0.032; *p* = 3.58 × 10^−8^). The severity of the 6OHDA lesion was anatomically assessed with *post hoc* immunostaining for TH, followed by unbiased stereological counts of TH^+^ neurons in the VTA and SNc. Lesioned animals showed significant cell loss in both the SNc and VTA when compared with their vehicle-injected counterparts [Fig. 5*D*,*E*; TH^+^ neuron ratio: vehicle SNc, 0.79 ± 0.049; 6OHDA SNc, 0.037 ± 0.015 (*p* = 1.31 × 10^−14^); vehicle VTA, 1.01 ± 0.063; 6OHDA VTA, 0.30 ± 0.048 (*p* = 3.32 × 10^−9^)], indicating that the unilateral injection had effectively depleted dopamine neurons and induced the expected motor impairment. Because the 6OHDA lesion caused a significant reduction of neurons in the VTA, we posited that this could lead to a reduction in TH^+^ boutons in M1, since M1 is a target of dopaminergic projections from the VTA and, to a lesser extent, the SNc (Hosp et al., 2011; Leemburg et al., 2018). To test this prediction, we performed stereological counts of TH^+^ boutons in L2/3 and L5 in a subset of lesioned and vehicle-injected animals where sections containing M1 had been collected and processed. Unilateral 6OHDA lesion of the SNc led to significant reduction of TH^+^ boutons in the ipsilateral M1 (Fig. 5*F*; vehicle L2/3, 1.14 ± 0.058; 6OHDA L2/3, 0.29 ± 0.034 (*p* = 0.0063); vehicle L5, 1.02 ± 0.067; 6OHDA L5, 0.31 ± 0.12 (*p* = 0.035)]. These data indicate that a nigral 6OHDA lesion is sufficient to reduce both midbrain dopaminergic cells as well as direct dopaminergic innervation of M1.

**Figure 6.**
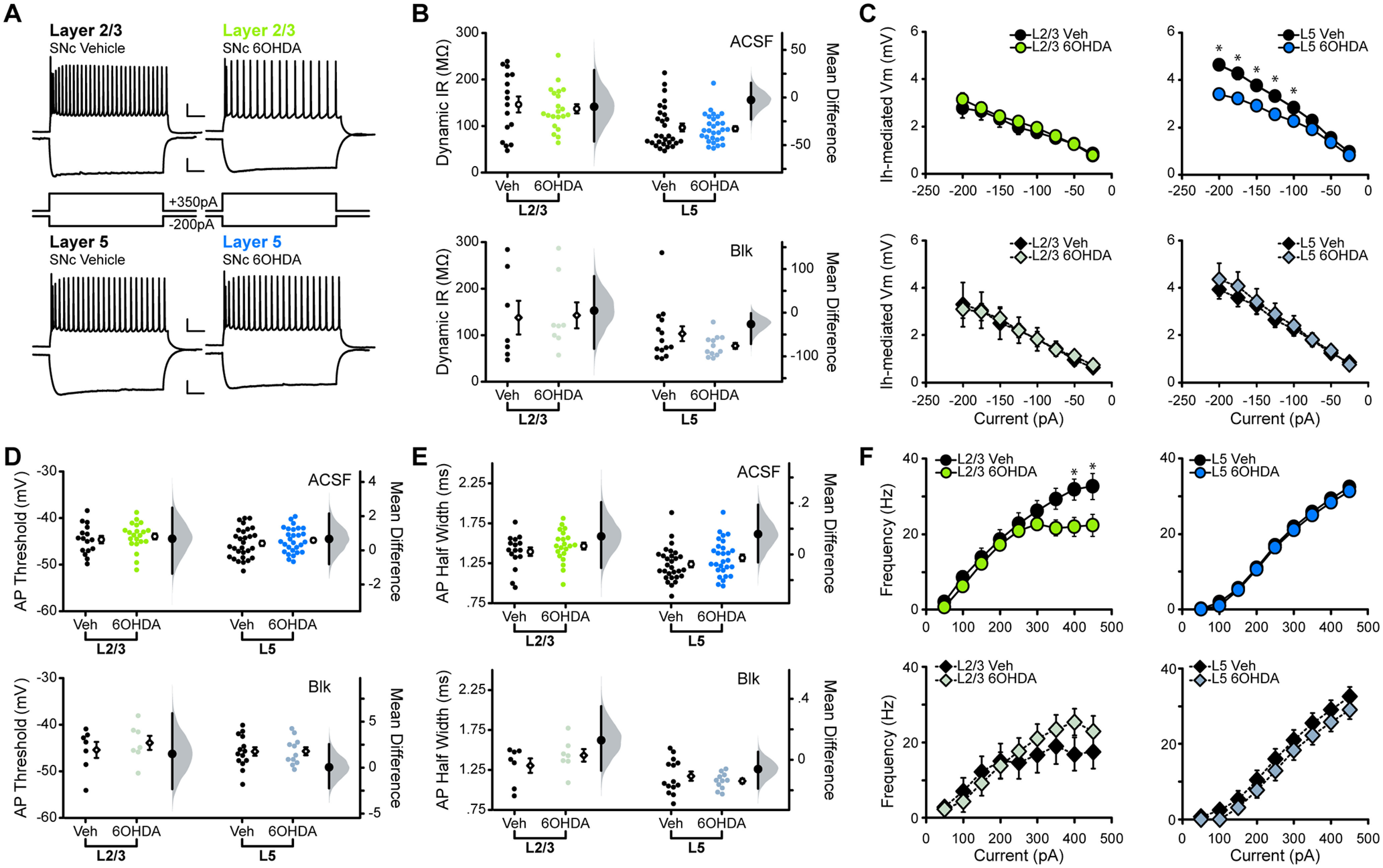
Nigral 6OHDA lesion shifts M1 neuron excitability, partially recapitulating the effects of D1R antagonist. ***A***, Superimposed responses to hyperpolarizing and depolarizing current steps in individual L2/3 (green) and L5 (blue) neurons of vehicle- and 6OHDA-injected animals. All traces shown are in ACSF conditions. Scale bar: Top, 20 mV, 100 ms; bottom, 10 mV, 100 ms. ***B–F***, Summary excitability plots for excitatory neurons in L2/3 and L5 of vehicle and 6OHDA animals, in baseline (ACSF) or synaptic blockers (BLK; 20 μm picrotoxin, 20 μm DNQX, 50 μm AP5) conditions. Modified Cumming plots show raw data of individual neurons as swarm plots, with the mean ± SEM offset to the right. Further right of each group of raw data are the effect size (black circle), corresponding 95% CIs (black vertical bars), and the underlying bootstrap sampling distribution. ***B***, Dynamic input resistance across hyperpolarizing current steps. ***C***, Voltage dependence of *I*_h_-mediated voltage sag elicited by hyperpolarizing current. ***D***, Action potential threshold at rheobase. ***E***, Action potential half-width at rheobase. ***F***, Action potential frequency during suprathreshold current injections. ACSF L2/3 vehicle neurons: *N* = 11, *n* = 16; ACSF L2/3 6OHDA neurons: *N* = 13, *n* = 21; BLK L2/3 vehicle neurons: *N* = 3, *n* = 7; BLK L2/3 6OHDA neurons: *N* = 4, *n* = 8; ACSF L5 vehicle neurons: *N* = 11, *n* = 29; ACSF L5 6OHDA neurons: *N* = 11, *n* = 29; BLK L5 vehicle neurons: *N* = 5, *n* = 14; BLK L5 6OHDA neurons: *N* = 6, *n* = 12. Data are shown as the mean ± SEM. *p ≤ 0.05.

We then assessed the effect of the 6OHDA manipulation on the input/output function of L2/3 and L5 pyramidal neurons in M1 (Fig. 6). In contrast to the acute blockade of D1R and D2R, chronic midbrain dopamine depletion did not alter input resistance, action potential threshold, or action potential half-width of either L2/3 or L5 neurons in M1 (Fig. 6*B*,*D*,*E*). Interestingly, the voltage sag was unaffected in L2/3 neurons, but significantly reduced in L5 (Fig. 6*C*). As acute D1R antagonism in L5 drove an increase in voltage sag, this indicates that the ablation of midbrain dopamine neurons engages mechanisms impacting M1 excitability that go beyond reduced activity of D1R. Further, this effect was eliminated when the experiment was performed in synaptic blockers, pointing to the involvement of synaptic mechanisms in the effect of chronic nigral dopamine depletion. These results were reinforced by estimation statistics performed on the data (Tables 1, 2), which together suggest that chronic loss of dopaminergic activity in the motor circuit does not fully recapitulate the effects of acute dopamine receptor blockade in M1.

Analysis of *f–I* curves for both L2/3 and L5 pyramidal neurons unveiled an effect similar to the effect of acute D1R blockade: L2/3 neurons of 6OHDA mice lost the ability to increase their firing rate at the highest current injections (Fig. 6*F*), and this effect was abolished by the application of synaptic blockers. The *f–I* curve of L5 neurons in 6OHDA mice was not significantly different from that of vehicle-injected mice in either ACSF or synaptic blockers (Fig. 6*F*). These results suggest that midbrain depletion of dopamine induces laminar-specific changes in the excitability of M1 pyramidal neurons, likely through a combination of altered dopaminergic signaling in M1 as well as through a shift in overall synaptic transmission in the area.

### Chronic depletion of dopaminergic input to M1 exclusively impacts L2/3 excitability

**Figure 7.**
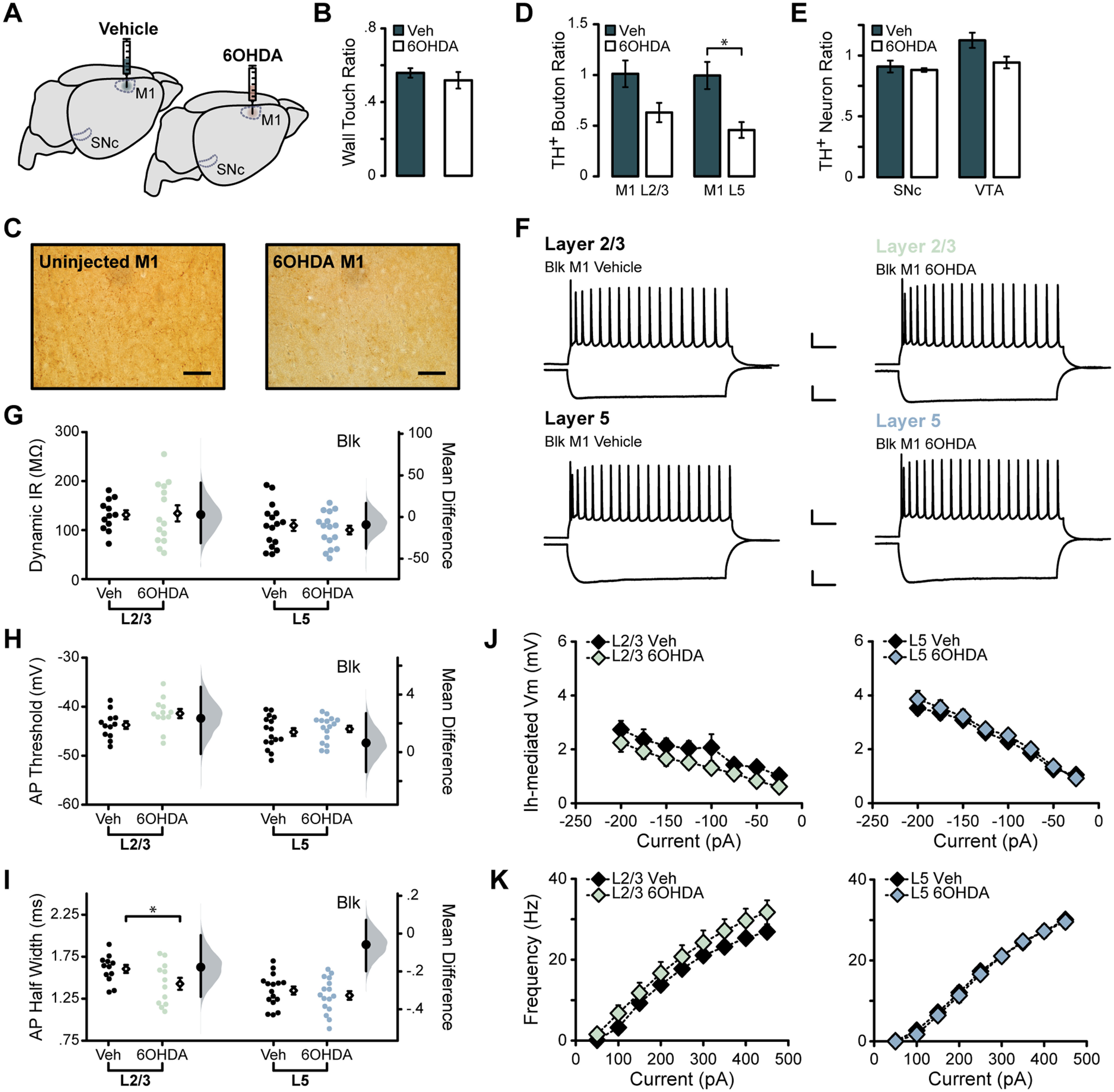
Chronic M1 dopamine depletion impacts L2/3 intrinsic excitability. ***A***, Unilateral injection of 6OHDA or vehicle into forelimb region of M1. ***B***, Quantification of weight-bearing wall touches in cylinder motor assessment. ***C***, TH^+^ axons and boutons labeled in L2/3 of M1 ipsilateral and contralateral to the injection site. Magnification, 40×. Scale bar, 50 μm. ***D***, Stereological counts of TH^+^ boutons in M1 in lesioned (*N* = 4) or vehicle-injected (*N* = 5) animals. ***E***, Stereological counts of TH^+^ neurons in the SNc and VTA of a subset of animals (vehicle, *N* = 4; 6OHDA, *N* = 4). ***F–K***, Summary excitability plots for excitatory neurons in L2/3 (green) and L5 (blue) of vehicle or 6OHDA-injected animals, performed in synaptic blockers (BLK: 20 μm picrotoxin, 20 μm DNQX, 50 μm AP5). Modified Cumming plots show raw data of individual neurons as swarm plots, with the mean ± SEM offset to the right. Further right of each group of raw data are the effect size (black circle), corresponding 95% CIs (black vertical bars), and the underlying bootstrap sampling distribution. ***F***, Superimposed responses to hyperpolarizing and depolarizing current steps in individual L2/3 and L5 neurons in vehicle- or 6OHDA-injected animals. Scale bar: Top, 20 mV, 100 ms; bottom, 10 mV, 100 ms. ***G***, Dynamic input resistance across hyperpolarizing current steps. ***H***, Action potential threshold at rheobase. ***I***, Action potential half-width at rheobase. ***J***, Voltage dependence of *I*_h_-mediated voltage sag elicited by hyperpolarizing current. ***K***, Action potential frequency during suprathreshold current injections. Vehicle L2/3 neurons: *N* = 4, *n* = 12; vehicle L5 neurons: *N* = 5, *n* = 16; 6OHDA L2/3 neurons: *N* = 4, *n* = 14; 6OHDA L5 neurons: *N* = 4, *n* = 16. Data are shown as the mean ± SEM. *p ≤ 0.05.

The nigral 6OHDA lesion primarily affected the SNc but also reduced the number of dopaminergic neurons in the VTA and TH^+^ boutons in M1. The effects of this manipulation only partially overlapped with those of acute dopamine receptors antagonism, leading us to investigate how the depletion of dopaminergic afferents exclusively in M1 would impact excitatory neuron excitability. Unilateral 6OHDA lesion of M1 did not induce motor impairment in the cylinder motor task compared with vehicle-injected controls [Fig. 7*B*; vehicle, 0.56 ± 0.026; 6OHDA, 0.52 ± 0.045 (*p* = 0.49)]. Stereological counts of TH^+^ boutons in M1 showed that the 6OHDA lesion significantly reduced TH^+^ boutons in ipsilateral L5 and reduction trending toward significance in ipsilateral L2/3 [Fig. 7*C*,*D*; vehicle L2/3, 1.012 ± 0.13; 6OHDA L2/3, 0.63 ± 0.10 (*p* = 0.068); vehicle L5, 1.00 ± 0.13; 6OHDA L5, 0.46 ± 0.079 (*p* = 0.017)], indicating that the 6OHDA injection was effective. In a subset of animals where midbrain tissue had been collected and processed, we performed stereological counts of TH^+^ neurons in the SNc and VTA to assess whether a 6OHDA lesion of M1 was sufficient to reduce dopaminergic neurons in the midbrain. Unilateral M1 6OHDA lesion had no impact on TH^+^ neurons in the ipsilateral SNc, while counts in the ipsilateral VTA showed a reduction in TH^+^ neurons that was trending toward significance [Fig. 7*E*; M1 vehicle SNc, 0.91 ± 0.070; M1 6OHDA SNc, 0.88 ± 0.052 (*p* = 0.76); M1 vehicle VTA, 1.12 ± 0.030; M1 6OHDA VTA, 0.94 ± 0.073 (*p* = 0.059)].

As the effects of acute D1R and D2R blockade were largely independent of synaptic transmission, patch-clamp recordings in M1 lesion experiments were conducted in the presence of synaptic blockers. L5 pyramidal neurons showed no differences in the subthreshold or suprathreshold range of activity (Fig. 7*F–K*, Table 2), suggesting that chronic loss of dopaminergic input to M1 did not affect the excitability of these neurons. In contrast, L2/3 neurons showed several changes following 6OHDA lesion in M1. First, the action potential threshold was depolarized, and statistical analysis of these data revealed that this effect was trending toward significance [Fig. 7*H*, Table 1; in mV: L2/3 vehicle, −43.74 ± 0.79; L2/3 6OHDA, −41.39 ± 0.93 (*p* = 0.067)], indicating that larger currents are required for these neurons to generate an action potential. Second, the action potential half-width of L2/3 neurons was significantly reduced following 6OHDA M1 lesioning [Fig. 7*I*, Table 1; in ms: L2/3 vehicle, 1.60 ± 0.048; L2/3 6OHDA, 1.43 ± 0.071 (*p* = 0.050)]. These shifts in L2/3 neuron excitability are specific to the M1 lesion experiment and are not recapitulated by either dopamine receptor blockade or midbrain dopaminergic cell loss. This suggests that local dopaminergic deafferentation within M1 causes unique changes in excitability specific to L2/3 pyramidal neurons. Together, our results indicate that dopamine impairment can have complex effects on the input/output function of M1 neurons depending on the duration and location of the dopamine impairment.

## Discussion

Dopaminergic signaling is crucial for skilled voluntary movement, and reduced dopamine in the motor circuit leads to motor impairment. PD is characterized by progressive dopaminergic cell death in the SNc, which primarily projects to the basal ganglia, and a less severe but significant loss of dopaminergic neurons in the VTA (Giguère et al., 2018). While the effects of reduced dopamine signaling has been well documented in the basal ganglia (Blandini et al., 2000; Day et al., 2006; Azdad et al., 2009; Bagetta et al., 2010; Fieblinger et al., 2014), how midbrain dopaminergic cell death affects M1 is less clear. Previous studies have highlighted the importance of dopamine in M1 for motor learning and plasticity (Molina-Luna et al., 2009; Rioult-Pedotti et al., 2015), and impairment of dopaminergic input to M1 *in vivo* results in impaired skill learning, delayed movement execution, and structural changes at M1 synapses (Hosp et al., 2011; Guo et al., 2015; Chen et al., 2019); however, the mechanisms underlying these changes are understudied. We demonstrate that impaired dopamine signaling impacts the excitability of M1 pyramidal neurons, an effect that could contribute to the diminished motor function observed in previous studies.

Acute D1R and D2R antagonism impacts the input/output function of M1 neurons, suggesting that dopamine regulates excitability in a healthy M1. Our results show that D1R blockade increases the excitability of L2/3 and L5 neurons in the subthreshold range of activity up to rheobase. Within the suprathreshold range of activity, on the other hand, layer-specific effects emerge. The *f–I* curve of L2/3 neurons was shifted downward following D1R antagonism, particularly impacting the response to large input. This effect could be driven by a depolarization block, which has been observed in hippocampal pyramidal neurons (Bianchi et al., 2012) and midbrain dopaminergic neurons (Canavier et al., 2016). In these instances, depolarization blocks have been ascribed to changes in kinetics and/or conductance of voltage-gated sodium channels (Qian et al., 2014) and delayed rectifier potassium channels (Bianchi et al., 2012). Other modeling studies investigating the currents that govern firing frequency and gain attribute the downshift to increased sodium conductance (Kispersky et al., 2012). In contrast, L5 neurons showed an upward shift in the linear portion of the *f–I* curve when fast synaptic transmission was blocked. This implies that D1R antagonism caused increased excitability of L5 neurons in their suprathreshold range as well; however, this effect was masked when spontaneous synaptic transmission was present. In fact, almost all effects of D1R antagonism persisted in the presence of fast synaptic transmission blockers, consistent with the direct expression of these receptors on the cells we recorded and with the engagement of intrinsic mechanisms for the modulation of membrane properties. The effects of D2R blockade were more subtle, although consistent with an overall increase in excitability in L2/3 and L5. These changes appear to be dependent on synaptic activity in L2/3, where the effects of D2R blockade were eliminated by synaptic transmission blockers. In contrast, many of the excitability shifts in L5 following D2R blockade, including an upward shift in the linear portion of the L5 *f–I* curve, persisted when synaptic transmission was blocked. The layer-specific differences in excitability following dopamine receptor blockade, particularly those that are synaptic transmission dependent, could be because of a differential and cell type-specific distribution of D1R and D2R expression. Effects that were abolished when synaptic transmission was blocked could be the result of D1R/D2R antagonism on other neurons, including inhibitory neurons, an effect that may shift incoming excitation or inhibition onto the recorded neurons in a manner that influences their excitability. Studies have shown that cortical inhibitory interneurons differentially express D1R and D2R subtypes (Le Moine and Gaspar, 1998; Anastasiades et al., 2019) and that increasing or decreasing their activity via dopaminergic modulation could in turn shift the excitability of neighboring pyramidal cells. Further studies are needed to examine the consequences of reduced dopamine signaling on interneurons in M1 and to understand their interaction with excitatory neurons in the same layer.

The increase in excitability following D1R blockade is surprising in view of studies in the basal ganglia showing that dopamine or D1R agonists typically increase excitability (Planert et al., 2013). However, reports show that dopamine, via D1R, reduces the excitability of L5 pyramidal neurons in the entorhinal cortex (Rosenkranz and Johnston, 2006). Additionally, dopamine application in M1 *in vivo* reduces spontaneous firing of corticospinal neurons (Huda et al., 2001; Awenowicz and Porter, 2002), which are most densely found in M1 L5 (Oswald et al., 2013). Analysis of neuronal excitability in experiments in which slices were preincubated in dopamine, and then a cocktail of dopamine and D1R and D2R antagonists, mimicked a transient shutdown of dopaminergic activity in M1 and induced shifts in excitability in accordance with these studies.

After establishing that the excitability of M1 pyramidal neurons is directly modulated by dopamine, we extended our study to assess how chronic loss of dopamine modulates the excitability of M1 pyramidal neurons. Loss of dopaminergic neurons projecting to the motor circuit, particularly to the basal ganglia, leads to movement dysfunction and is a hallmark of PD. Chronic depletion of dopamine impacts neural activity across the motor circuit (Blandini et al., 2000; Day et al., 2006; Azdad et al., 2009; Planert et al., 2013; Benazzouz et al., 2014). We posited that chronic loss of dopamine to all areas involved in motor control would lead to reverberating changes in excitability within M1, despite this region receiving only a fraction of the dopaminergic input. In agreement with previous work, our 6OHDA-injected mice showed unilaterally impaired forelimb use. Furthermore, 6OHDA mice showed laminar-specific shifts in excitability within M1, indicating that neurons in L2/3 and L5 have distinct sensitivity to chronic loss of dopamine signaling. Contrasting the effects of acute D1R blockade, L2/3 neurons in 6OHDA mice showed no change in the subthreshold range of activity. However, the *f–I* curve of L2/3 neurons showed impaired response to large inputs, mirroring the effect of acute D1R antagonism. This change was eliminated by synaptic transmission blockers, suggesting that, unlike the effect of acute D1R blockade, this comparable shift in the *f–I* curve of L2/3 neurons is dependent on synaptic transmission. The excitability of L5 neurons was also affected by chronic dopamine depletion, although the effects were unique to this cortical layer and dopamine manipulation. Chronic midbrain dopamine depletion induced a decrease in *I*_h_ of L5 cells with no changes in the suprathreshold range of their input/output function. The decrease in *I*_h_ was fully eliminated by the application of fast synaptic transmission blockers, suggesting that it is mediated by synaptic activity. Decreased *I*_h_ alters the capacity of the neuron to maintain resting membrane potential and impacts the response to input, particularly those that would hyperpolarize the cell.

Overall, the changes in excitability because of midbrain dopamine loss rely on synaptic drive, suggesting that chronic midbrain dopamine manipulation may alter fast synaptic transmission into or within M1. This could occur as a consequence of shifting activity within the basal ganglia following dopamine loss, as an effect of reduced dopamine signaling directly in M1, or a combination of these factors. While a thorough analysis of synaptic transmission is beyond the scope of this study, there are reports of altered synaptic activity in models of PD elsewhere in the motor circuit (Day et al., 2006; Fan et al., 2012; Galvan et al., 2015; Parker et al., 2016), pointing to the possibility that the excitatory drive of M1 may be reduced.

As the effects of a nigral 6OHDA lesion only partially overlapped with those observed using dopamine receptor antagonists, we predicted that these changes to M1 pyramidal neuron input/output function were likely because of altered synaptic activity across the motor circuit rather than simply from the loss of dopaminergic input exclusively to M1. In support of this interpretation, restricting the 6OHDA injection to M1 altered the excitability of L2/3, but not L5, pyramidal neurons with apparently opposing effects to those caused by midbrain dopamine depletion or acute D1R and D2R blockade. The action potential threshold of L2/3 pyramidal neurons was depolarized and the half-width was decreased, indicating a shift in the currents governing action potential dynamics around rheobase. These results differ from those obtained following all other manipulations. We speculate that they result from a combinatorial, chronic reduction in M1 D1R and D2R activity on all neuron types and may result from compensatory mechanisms following the chronic local loss of dopaminergic innervation. Dopamine depletion in M1 did not induce motor impairment, pointing to a limited impact of direct dopaminergic projections in motor execution. Such a result is expected, as direct dopamine modulation in M1 is primarily associated with synaptic plasticity and motor learning, aspects that we did not examine (Molina-Luna et al., 2009; Hosp et al., 2011).

### Functional implications

Dopamine receptor blockade increased the excitability of L2/3 and L5 neurons. In M1, L2/3 pyramidal neurons are mainly corticocortical or corticostriatal projecting, while those in L5 are corticospinal, corticothalamic, and corticostriatal projecting (Oswald et al., 2013). Signals flow superficial to deep, with high intracortical connectivity between L2/3 neurons and corticospinal neurons in L5 (Weiler et al., 2008). Our results show that acute impairment of D1R and D2R signaling increases the excitability of L5 neurons along with the excitability of one of their primary presynaptic partners. Together, these changes may lead to hyperactivity in M1, an effect associated with motor impairment and movement disorders like PD (Thobois et al., 2000; Haslinger et al., 2001).

Our results also show that acute dopamine receptor antagonism increased the *I*_h_-mediated sag in L2/3 and L5, while chronic dopamine loss by midbrain 6OHDA injections decreased the sag selectively in L5. In M1, *I*_h_ is thought to be a regulator of signal flow from superficial layers, which is involved in motor planning, to deeper output layers (Sheets et al., 2011). We show that dopamine can directly and indirectly influence the activity of *I*_h_, providing evidence for one source of neuromodulatory control of signal flow in M1. Interestingly, one study of an animal model of PD reported downregulation of HCN2 channels, which in part mediate *I*_h_, in the globus pallidus (Chan et al., 2011). This led to abolished autonomous pacemaking activity and induced abnormal synchronous activity. While reintroduction of HCN2 channels in the globus pallidus restored normal signaling, it was not sufficient to recover motor impairments. In view of our findings, one may speculate that abnormal HCN activity in PD may extend beyond the globus pallidus, and that coordinated restoration of the activity of these channels may be required for symptom improvement.

### Conclusions

Dopamine signaling in the motor system is crucial for the execution of voluntary movements. While most work focuses on the effects of dopamine signaling in the basal ganglia, recent studies point to M1 as an additional site of dysfunction in PD patients and mouse models of the disease. Our results indicate that diminished dopamine signaling, whether acute or chronic, has profound effects on the excitability of M1 neurons. We unveil a complex combination of laminar-specific mechanisms for dopamine-dependent modulation of pyramidal neuron excitability, which are likely to significantly alter the output of M1 and influence movement execution.

## Synthesis

Reviewing Editor: Mikko Airavaara, University of Helsinki Decisions are customarily a result of the Reviewing Editor and the peer reviewers coming together and discussing their recommendations until a consensus is reached. When revisions are invited, a fact-based synthesis statement explaining their decision and outlining what is needed to prepare a revision will be listed below. The following reviewer(s) agreed to reveal their identity: Jun Ding. Note: If this manuscript was transferred from JNeurosci and a decision was made to accept the manuscript without peer review, a brief statement to this effect will instead be what is listed below.

The reviewers fully agree on the scientific merit of your study. As you will see below, they also have a number of suggestions, including additional experiments, which will only strengthen your conclusions. Note that they also raised some concerns regarding

Reviewer 1:

In this study the authors investigated the direct and synaptically-mediated effect of dopamine on input/output function of layer 2/3 and layer 5 primary motor cortex (M1) neurons. In order to decipher the respective contribution of fast synaptic transmission and direct intrinsic properties changes in the dopaminergic effects on pyramidal neurons, the authors performed electrophysiological recordings in the presence or in the absence of fast synaptic transmission blockers.

The role of dopamine transmission on pyramidal neurons was assessed by two strategies: first, they used D1 and D2 receptor antagonists and second they studied the effect of dopaminergic denervation. For this second strategy, the authors used two approaches: (1) denervation of cortical and subcortical motor regions (by SNc 6-OHDA injection inducing SNc and partial VTA TH positive cells loss) and (2) a dopaminergic denervation restricted to M1 (by 6-OHDA injected locally). The results demonstrate that D1 antagonist SCH23390 increased excitability through modulation of intrinsic properties, while D2 antagonist sulpiride depended on synaptic transmission. VTA and SNc loss of dopamine neurons also induced synapse-dependent modulation of M1 neurons input/output function. The authors also show that dopamine receptor antagonism increased the Ih-mediated sag in both layers while midbrain dopamine denervation decreased the sag selectively in L5. Eventually, dopamine denervation in M1 induced an increase in layer 2/3 neurons excitability but not in layer 5. This effect was not observed in

dopamine receptor blockade or midbrain dopaminergic cell loss experiments.

The work achieved by the authors is commendable, investigating the impact of dopamine transmission on M1 pyramidal neurons in multiple layers, using different readouts of pre- and post-synaptic changes and using multiple approaches from pharmacology to lesion. The manuscript is well written and all experimental procedures are comprehensively described.

Major comments:

1) The authors investigate acute loss of dopamine signaling using SCH23390 and sulpiride as D1 and D2 receptor antagonists respectively. However, no basal dopamine tone is expected in a slice preparation, and both antagonists are known to act as inverse agonists (Roberts DJ and Strange PG, 2005; Cai G. et al, 1999). Thus the effect of this pharmacological treatments might not account for an interruption of dopamine-induced D1 or D2 signaling but may more likely be due to an effect of both compounds as inverse agonists. To conclude on the acute loss of dopamine signaling it is necessary to compare the effect of dopamine bath application with the effect of dopamine + D1 or D2 antagonists.

2) Since dopamine cell loss is observed in the VTA following SNC 6-OHDA treatment, it would be interesting to show TH immunohistochemistry in M1 and to quantify TH button in this region. Indeed, Hosp et al. (2011) demonstrated that 6-OHDA lesion in the VTA induced dramatic TH denervation in M1. This information would allow a more accurate comparison of results obtained after midbrain or M1 dopamine depletion.

3) Following 6-OHDA injection in M1, dopamine cell loss in the midbrain is expected. It would be relevant to quantify this dopamine cell loss both in the VTA and SNc.

Reviewer 2:

The authors provide a novel account of layer-specific changes in excitability resulting from dopamine neuron ablation and receptor antagonism in the primary motor cortex. The divergent findings between acute antagonism, SNc ablation, and M1 ablation are striking and are interesting to consider within the context of Parkinson’s model. However, there are several issues pertaining to the analysis and interpretation of certain experiments:

Major concerns:

• In figure 2 panel F, the authors observe that the max firing rate in D1 antagonist treated cells decreases at larger current injection steps. By contrast, the maximum firing rate for cells in panels 5F and 6F reaches a plateau. Could the authors please elaborate as to why they observe lower firing rates at higher steps? The authors do not mention any criteria used to assess whether a cell was in depolarization block and should analyze separately cells that exhibit blockade.

• Related to the previous point, in figure 2 panel D, the authors find that D1 antagonism significantly lowers the action potential threshold (consistent with increased excitability) but in panel F they observe that D1 receptor antagonism lowers the firing rate (consistent reduced excitability). Could authors elaborate as to the mechanism that may account for these divergent findings?

• Conversely in figure 6 the authors observe increased action potential threshold (less excitable), but also increased firing rates in 6OHDA lesioned mice. Again, could the authors please comment on why they observe these divergent findings?

• In the statistics section, the authors state the data were analyzed with either Student’s t-tests or Wilcoxon/Mann-Whitney U-tests. However, these tests rely on independent samples and the authors do not state that any other types of analyses were performed. This poses a problem for the interpretation of Input-Output functions where Ih currents and firing rates are compared within the same cells. Due to the number of comparisons being made, the data should be analyzed with an ANOVA and post-hoc tests (eg. Tukey’s HSD) to determine which specific current steps are different between groups. It is possible that the authors may have performed this type of analysis, however because degrees of freedom are not reported, it is difficult to discern what analyses are being performed.

Minor concerns:

• Line 396 “In both layers this effect was eliminated by in the presence of...” the authors should omit the word “by”.

• In line 443-444 the authors state that “... but significantly reduced in L5, which opposes the effect observed following acute D1R antagonism”. Since the effects of D1R antagonism were not performed in the 6OHDA model, the use of the word opposes here is confusing as it is not certain that the same drug effects would be observed in the Parkinson’s model. It would be preferable if the authors stated that the results were “in contrast to” the results observed with acute pharmacology.

STATS: In the statistics section, the authors state the data were analyzed with either Student’s t-tests or Wilcoxon/Mann-Whitney U-tests. However, these tests rely on independent samples and the authors do not state that any other types of analyses were performed. This poses a problem for the interpretation of Input-Output functions where Ih currents and firing rates are compared within the same cells. Due to the number of comparisons being made, the data should be analyzed with an ANOVA and post-hoc tests to determine which specific current steps are different between groups. It is possible that the authors may have performed this type of analysis, however because degrees of freedom are not reported, it is difficult to discern what analyses are being performed.

## Author Response

Editors @ eNeuro

We wish to thank the Editor and the reviewers for their valuable feedback and suggestions. We have added a new experiment in which we confirmed the effect of dopamine receptor blockers by bath application of the receptor antagonists following the establishment of a dopamine tone in our slice preparation. These data are now reported in a new figure. We also increased sampling for experiments aimed at investigating the effect of local, chronic dopamine depletion within M1. The entire data set was reanalyzed using estimation statistics. Finally, we added additional stereology counts of dopamine neurons and putative dopaminergic puncta as suggested by the reviewers. The manuscript text has been extensively edited to include the new procedures, data and analyses, as well as the clarifications requested. We feel that the manuscript has been greatly improved by the revision.

A detailed response to the reviewers’ concerns is provided below.

Reviewer 1:

The work achieved by the authors is commendable, investigating the impact of dopamine transmission on M1 pyramidal neurons in multiple layers, using different readouts of pre- and post-synaptic changes and using multiple approaches from pharmacology to lesion. The manuscript is well written and all experimental procedures are comprehensively described.

We are thankful that the reviewer found our study commendable.

Major comments:

1-The authors investigate acute loss of dopamine signaling using SCH23390 and sulpiride as D1 and D2 receptor antagonists respectively. However, no basal dopamine tone is expected in a slice preparation, and both antagonists are known to act as inverse agonists (Roberts DJ and Strange PG, 2005; Cai G. et al, 1999). Thus the effect of this pharmacological treatments might not account for an interruption of dopamine-induced D1 or D2 signaling but may more likely be due to an effect of both compounds as inverse agonists. To conclude on the acute loss of dopamine signaling it is necessary to compare the effect of dopamine bath application with the effect of dopamine + D1 or D2 antagonists.

To address this concern, we performed a new set of experiments during which we first bath applied dopamine-ACSF to provide a dopamine tone to the slice, then simultaneously bath applied the D1R and D2R antagonists in a dopamine-ACSF solution. Each solution contained the same concentration of DMSO to rule out any changes in excitability due to an increase in drug solvent. The rationale for applying a cocktail of D1R and D2R antagonists was the following: Firstly, the combined antagonist condition more closely approximates the effect of diminished dopamine axons in M1 following 6OHDA lesion. Secondly, as we observed shifts in excitability with the same sign with either D1R or D2R blockers, the compound application allowed us to assess possible additive effects of the antagonists in the presence of dopamine tone. Details of the experimental design from this new data set are now reported in the Methods (lines 195-199; 209-215) and Results (lines 444-474), and in the newly added Figure 4.

2) Since dopamine cell loss is observed in the VTA following SNC 6-OHDA treatment, it would be interesting to show TH immunohistochemistry in M1 and to quantify TH button in this region. Indeed, Hosp et al. (2011) demonstrated that 6-OHDA lesion in the VTA induced dramatic TH denervation in M1. This information would allow a more accurate comparison of results obtained after midbrain or M1 dopamine depletion.

We have stereologically quantified TH+, putative dopaminergic boutons in L2/3 and L5 of M1 following injections of 6OHDA in the SNc in a subset of animals as suggested. Results are now reported in Figure 5F and lines 497-506. Nigral 6OHDA lesion led to a significant decrease in TH+ boutons both in L2/3 and L5 of ipsilateral M1.

3) Following 6-OHDA injection in M1, dopamine cell loss in the midbrain is expected. It would be relevant to quantify this dopamine cell loss both in the VTA and SNc.

We have stereologically quantified TH+ positive cell bodies in VTA and SNc is a subset of M1-lesioned or control animals. Results are now reported in Figure 7E and in lines 539-545. We did not observe changes in the number of TH+ cell bodies in SNc, but in the VTA the reduction of TH+ cell bodies was trending toward significance (p = 0.059).

Reviewer 2:

The authors provide a novel account of layer-specific changes in excitability resulting from dopamine neuron ablation and receptor antagonism in the primary motor cortex. The divergent findings between acute antagonism, SNc ablation, and M1 ablation are striking and are interesting to consider within the context of Parkinson’s model.

We are thankful that the reviewer found our results interesting.

Major concerns:

• In figure 2 panel F, the authors observe that the max firing rate in D1 antagonist treated cells decreases at larger current injection steps. By contrast, the maximum firing rate for cells in panels 5F and 6F reaches a plateau. Could the authors please elaborate as to why they observe lower firing rates at higher steps? The authors do not mention any criteria used to assess whether a cell was in depolarization block and should analyze separately cells that exhibit blockade.

We understand the concern of the reviewer. However, we did not directly compare fI curves between cells in D1R blockade, cells from SNc 6OHDA animals, and cells from M1 6OHDA animals. Our analysis focused on comparisons within the test groups for each experiment. As any chronic reduction in dopaminergic neurons or their afferents would impact all dopamine receptor subtypes, it would be necessary to design specific experiments that go beyond the scope of this study so that rigorous direct comparisons between conditions could be made. Nevertheless, we first addressed this concern by adding n to the vehicle injected animals in the M1 lesion experiments so that the group was a more comparable sample size to the lesioned condition and other experiments. These data are shown in Figure 7, and there is now no significant difference between L2/3 fI curves in this experiment. Further, we discussed depolarization block in our fI curves in the Discussion (Lines 583-589).

• Related to the previous point, in figure 2 panel D, the authors find that D1 antagonism significantly lowers the action potential threshold (consistent with increased excitability) but in panel F they observe that D1 receptor antagonism lowers the firing rate (consistent reduced excitability). Could authors elaborate as to the mechanism that may account for these divergent findings?

The issue raised by the reviewer is very interesting. Indeed, we observe an increase in excitability in the subthreshold-rheobase range. However, we did not observe a significant shift in the linear portion of the fI curve. The decreased firing rate at the larger depolarization steps suggests that loss of dopamine signaling decreases the range in which neurons can reliably fire action potentials. Such an effect may be due to depolarization block, as suggested by the reviewer and now speculated in the Discussion. The mechanisms underlying depolarization block are incompletely understood. In our data set, as the action potential half width increases and firing rates drop at high depolarizing steps, it is tempting to speculate that the drop in firing rate may be due to decreased function of delayed rectifier conductance. However, additional ion channels may contribute to the inability of neurons to fire in response to the larger depolarization steps. Investigation of all possible conductance underlying this effect requires a large body of experiments that goes beyond the scope of this study.

• Conversely in figure 6 the authors observe increased action potential threshold (less excitable), but also increased firing rates in 6OHDA lesioned mice. Again, could the authors please comment on why they observe these divergent findings?

As mentioned above, we realized that the sampling for this data set was smaller compared to the M1 lesioned group, as well as the other experiments, so before speculating on possible mechanisms we repeated and expanded this data set. Once the new animals and neurons were added to the analysis, the depolarization of the action potential threshold remained significant, while the effect of M1 lesion on the fI curve did not hold. We now report the new data set in Fig 7 and its reanalysis using estimation statistics.

The depolarization of action potential threshold is unique to the M1 lesion. In all other cases in our data where we report an increase in the linear portion of the fI curve (L5, Fig 2F and Fig 3F), those neurons also show a hyperpolarization of the action potential threshold. These two effect are consistent with one another.

• In the statistics section, the authors state the data were analyzed with either Student’s t-tests or Wilcoxon/Mann-Whitney U-tests. However, these tests rely on independent samples and the authors do not state that any other types of analyses were performed. This poses a problem for the interpretation of Input-Output functions where Ih currents and firing rates are compared within the same cells. Due to the number of comparisons being made, the data should be analyzed with an ANOVA and post-hoc tests (eg. Tukey’s HSD) to determine which specific current steps are different between groups. It is possible that the authors may have performed this type of analysis, however because degrees of freedom are not reported, it is difficult to discern what analyses are being performed.

We have expanded the description of our statistical analysis procedures in the Methods section to provide clarity and to outline the use of estimation statistics. These additions can be found in the Methods (lines 298-320) and in Table 1 and 2.

Minor concerns:

• Line 396 “In both layers this effect was eliminated by in the presence of...” the authors should omit the word “by”.

Corrected. Thank you.

• In line 443-444 the authors state that “... but significantly reduced in L5, which opposes the effect observed following acute D1R antagonism”. Since the effects of D1R antagonism were not performed in the 6OHDA model, the use of the word opposes here is confusing as it is not certain that the same drug effects would be observed in the Parkinson’s model. It would be preferable if the authors stated that the results were “in contrast to” the results observed with acute pharmacology.

The sentence has been edited to clarify this issue. Lines 510-514.
